# Isocyanide-based multicomponent reactions towards cyclic constrained peptidomimetics

**DOI:** 10.3762/bjoc.10.50

**Published:** 2014-03-04

**Authors:** Gijs Koopmanschap, Eelco Ruijter, Romano VA Orru

**Affiliations:** 1 Department of Chemistry & Pharmaceutical Sciences, Amsterdam Institute of Molecules, Medicines and Systems, VU University Amsterdam, de Boelelaan 1083, 1081 HV, Amsterdam, The Netherlands

**Keywords:** heterocycles, isocyanides, macrocycles, multicomponent reaction, medicinal chemistry, organic synthesis, peptidomimetics

## Abstract

In the recent past, the design and synthesis of peptide mimics (peptidomimetics) has received much attention. This because they have shown in many cases enhanced pharmacological properties over their natural peptide analogues. In particular, the incorporation of cyclic constructs into peptides is of high interest as they reduce the flexibility of the peptide enhancing often affinity for a certain receptor. Moreover, these cyclic mimics force the molecule into a well-defined secondary structure. Constraint structural and conformational features are often found in biological active peptides. For the synthesis of cyclic constrained peptidomimetics usually a sequence of multiple reactions has been applied, which makes it difficult to easily introduce structural diversity necessary for fine tuning the biological activity. A promising approach to tackle this problem is the use of multicomponent reactions (MCRs), because they can introduce both structural diversity and molecular complexity in only one step. Among the MCRs, the isocyanide-based multicomponent reactions (IMCRs) are most relevant for the synthesis of peptidomimetics because they provide peptide-like products. However, these IMCRs usually give linear products and in order to obtain cyclic constrained peptidomimetics, the acyclic products have to be cyclized via additional cyclization strategies. This is possible via incorporation of bifunctional substrates into the initial IMCR. Examples of such bifunctional groups are *N*-protected amino acids, convertible isocyanides or MCR-components that bear an additional alkene, alkyne or azide moiety and can be cyclized via either a deprotection–cyclization strategy, a ring-closing metathesis, a 1,3-dipolar cycloaddition or even via a sequence of multiple multicomponent reactions. The sequential IMCR-cyclization reactions can afford small cyclic peptide mimics (ranging from four- to seven-membered rings), medium-sized cyclic constructs or peptidic macrocycles (>12 membered rings). This review describes the developments since 2002 of IMCRs-cyclization strategies towards a wide variety of small cyclic mimics, medium sized cyclic constructs and macrocyclic peptidomimetics.

## Introduction

Peptides and proteins fulfill a key role in many biological and physiological functions of living organisms. Therefore, they are interesting starting points for the development of novel drugs [1–2]. Peptides may act as neurotransmitters, hormones or antibodies and are involved in the progress of several diseases. However, natural peptides and proteins possess several properties which make them less suitable as a drug candidate. First, the amide bond is easily cleaved by proteases and their hydrophilic character results in a low permeability, rapid metabolic processing and excretion. Also, natural peptides often occur in an ensemble of conformations thereby reducing their specificity for biological targets resulting in unwanted side effects [3–4]. Consequently, chemists started to develop so-called peptidomimetics; *compounds that mimic the action or active conformation of a peptide by incorporating non-peptidic structural and functional features that imitate those of the parent peptide but with improved biological properties*. During the last decades, several classes of peptidomimetics have been described such as peptide bond isosteres or conformational constraint mimics [5–6]. Insertion of conformational restrictions is of high interest since they reduce the number of conformations, which may result in higher affinity for the target/receptor and improved protease stability, bioavailability and specificity [6–8]. Conformational bias can be achieved via *N*-alkylation, α-alkylation or the introduction of alkene amide bond isosteres, but also via local or global cyclization. A prominent advantage of cyclized constraints is that they force the molecule into a well-defined secondary structure. Such structural features are often found in biologically active peptides and proteins [8]. Mimicking the secondary structure is of high interest since these motifs are regularly located at the surface of peptide–peptide interactions [9]. Another important reason for the design and synthesis of these cyclic mimics is that they can give in-depth insight in the biologically active conformation of a peptide or protein [10].

In the context of design and synthesis of peptidomimetics, several approaches have been applied and most of them include a sequence of multiple reactions along with a variety of protection and deprotection steps. However, via these rather long sequential procedures it is often not straightforward to introduce structural diversity in a set of targeted peptide mimics, which is essential for effective fine tuning of biological activity [11–13]. Therefore, the use of more straightforward and robust reactions that can introduce complexity and structural diversity in only a few steps is highly desirable. A promising approach that combines those features is the use of multicomponent reactions (MCRs). Multicomponent reactions are convergent one-pot transformations involving three or more substrates that give a single product with high atom-economy. The reagents herein react in a sequential manner and all intermediate-steps are in equilibrium except often the last irreversible step, which provides the product. Besides saving time and reagents, another major advantage of these reactions is the ability to combine commercially available or readily accessible starting materials with a variety of functionalities in one-step. Further, MCR-based strategies can cover a broader range of chemical space because a large set of structurally different starting materials is tolerated and structural diversity is relatively easily achieved. Finally, the highly convergent nature of MCRs results in the generation of highly complex structures in only one step. In addition, because of their practical simplicity they are also ideally suited for automated synthesis [14–18]. Among the MCRs, the isocyanide-based multicomponent reactions (IMCRs), such as the Ugi and the Passerini reaction, are the most relevant reactions for constructing peptidomimetics since they give access to (depsi)peptide-like structures. However, these IMCRs provide linear products, whereas their cyclic analogues are highly desirable as discussed above for potentially improved structural and biological properties. Fortunately, these linear products can be cyclized via post-condensation transformations since a wide range of unreactive functional groups are tolerated in the IMCRs [19].

In this overview we focus on all recent developments in the last decade in the field of cyclic peptidomimetics obtained from IMCRs and their subsequent cyclization reactions. The cyclic mimics herein range from small rings (four to seven membered), medium sized rings (9–12 membered) to macrocycles.

### Isocyanide-based multicomponent reactions for cyclic peptidomimetics

Multicomponent reactions that include isocyanide or isocyanide derivatives (e.g. isocyanoacetates) have been widely applied for the synthesis of peptidomimetics. The main advantage of these isocyanide-based reactions is that the isocyanide functionality can act both as nucleophile and electrophile at the C1-carbon, which makes the construction of linear peptide-like structures possible [20]. Cyclic peptidomimetics can be obtained via subsequent transformations that in turn are possible via e.g. the incorporation of bifunctional substrates or by activation of functionalized substrates in the initial MCR [19].

### The Passerini reaction

**Scheme 1:**
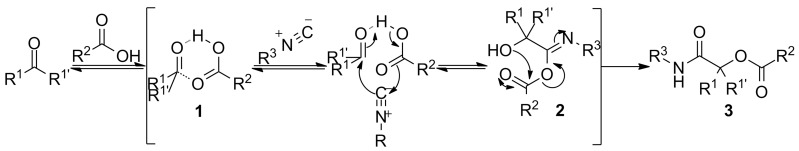
The proposed mechanism of the Passerini reaction.

The first isocyanide-based MCR was described by Mario Passerini in 1921 and named after him. The Passerini reaction is a three-component reaction (3-CR) and provides α-acyloxy carboxamides by reacting carbonyl compounds, carboxylic acids and isocyanides. The reaction is usually performed with high concentrations of starting materials using aprotic solvents. A wide range of all three components is tolerated in the Passerini 3-CR, which makes this reaction ideally suited for addressing scaffold diversity. The higher rates observed in aprotic solvents suggest that the Passerini 3-CR proceeds via a non-ionic pathway. A generally accepted mechanism starts with the generation of the loosely hydrogen-bonded adduct **1** from the oxo-component and the carboxylic acid (Scheme 1). The next step involves the α-addition of both the electrophilic carbonyl-carbon of the oxo-component and the nucleophilic oxygen of the acid component to the isocyanide, to afford the α-adduct **2**. A subsequent rearrangement then provides the α-acyloxy amide **3** [20–22].

**Scheme 2:**
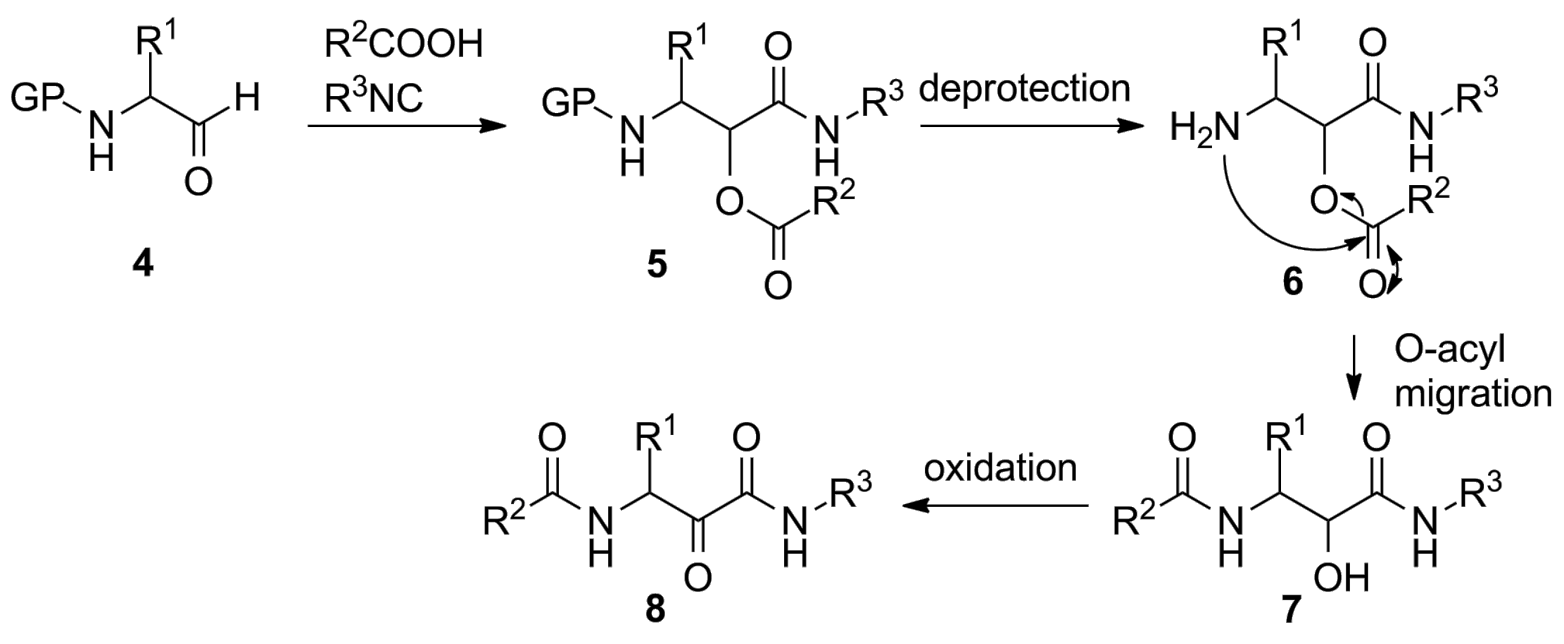
The PADAM-strategy to α-hydroxy-β-amino amide derivatives **7**. An additional oxidation provides α-keto amides **8**.

With regard to peptidomimetic design, the incorporation of *N*-protected aldehydes **4** (Scheme 2) is of great importance since deprotection of the α-adduct **5** allows acyl-migration and give access to α-hydroxy-β-amino amide derivatives **7** that possess important biological properties. This Passerini–amine deprotection–acyl migration (PADAM) strategy was reported for the first time by Banfi and co-workers in 2003 [23]. In addition, subsequent oxidation of **7** gives access to α-keto amides **8** that show important protease inhibitory activities.

### The Ugi reaction

**Scheme 3:**
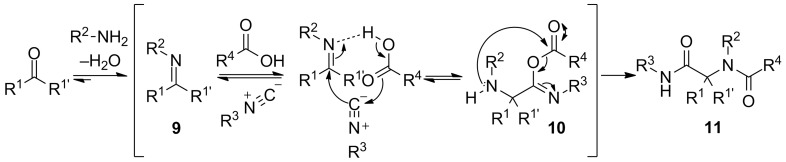
The general accepted Ugi-mechanism.

One of the most important MCRs that generates peptide-like structures was reported for the first time by Ivar Ugi in 1959. This Ugi four-component reaction (U-4CR) furnishes α-acylamino amides **11** by combining oxo-substrates, carboxylic acids, amines and isocyanides in one-pot and like the Passerini reaction a wide variety of substrates is tolerated. In contrast to the Passerini 3-CR, the Ugi 4-CR is favoured in polar protic solvents like low-molecular weight alcohols such as methanol, ethanol or trifluoroethanol. However, many examples in polar aprotic solvents are also reported. The generally accepted mechanism for the Ugi reaction proceeds via in situ imine formation of **9**, followed by the generation of α-adduct **10** formed via an attack of the isocyanide to the imine and a subsequent attack of the carboxylate to the resulting nitrilium ion (Scheme 3). The final dipeptide-like product is formed via a subsequent Mumm rearrangement of the α-adduct **10**. In addition, pre-formation of the imine or the use of bifunctional inputs (e.g. amino acids) can reduce this Ugi-4CR to an Ugi-3CR. In particular, the Ugi reaction with bifunctional inputs is called an Ugi-four-center-three-component reaction (U-4C-3CR) and has been extensively applied in peptidomimetic synthesis [21–22,24–25].

### Post-condensation strategies

**Scheme 4:**
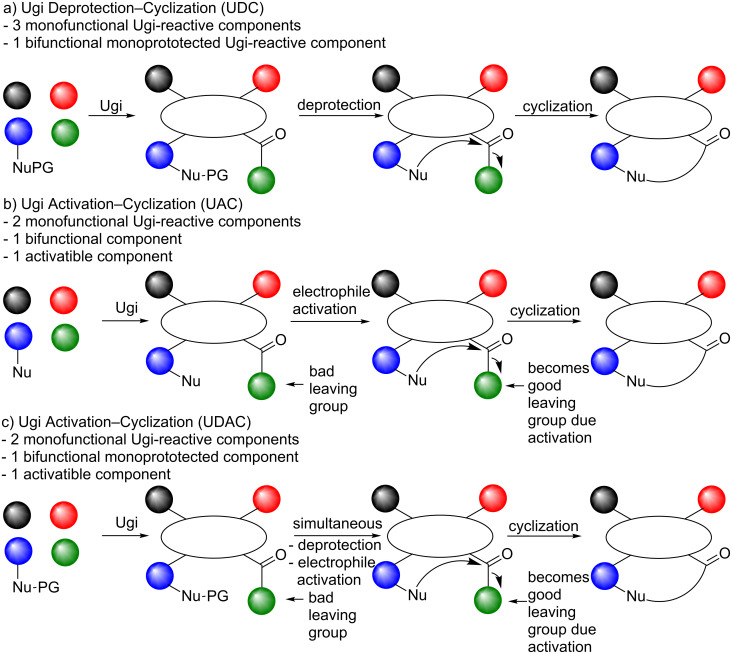
Three commonly applied Ugi/cyclization approaches. a) UDC-process, b) UAC-sequence, c) UDAC-combination.

**Scheme 5:**
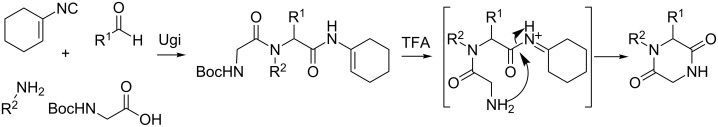
Ugi reaction that involves the condensation of Armstrong’s convertible isocyanide.

In the last decade, several peptidomimetics containing four to seven membered rings (including bicyclic systems), medium sized rings and macrocyclic systems have been reported via IMCRs. However, as both the Ugi and Passerini reactions provide linear products, several cyclization strategies have been utilized in order to obtain cyclic constructs. For example, the incorporation of cyclic imines immediately gives cyclic MCR-products, whereas other strategies make use of unreactive, convertible or protected functional Ugi-substrates that can be cyclized via subsequent transformations [19,22,26]. Examples of IMCR orthogonal species or functionalities are alkenes, alkynes and azides which may give the cyclic analogues via subsequent ring closing metathesis (RCM) or 1,3-dipolar cycloadditions. In contrast, protected functional groups first require a deprotection before a follow-up cyclization event can take place. An example of this is the use of *N*-Boc protected amino acids in the Ugi reaction. Subsequent deprotection of the linear Ugi-product allows cyclization and reactions of this type are classified as Ugi Deprotection–Cyclizations (UDC, Scheme 4). Moreover, cyclizations can also be initiated by activation of the Ugi-product via an Ugi Activation–Cyclization procedure and involves the use of convertible isocyanides as Ugi-substrates. An example of a convertible isonitrile is Armstrong’s isocyanide which can be cleaved after acidic treatment (Scheme 5). A combination of deprotection and activation is also possible and is found in the literature as an Ugi Deprotection/Activation–Cyclisation (UDAC). In addition, other MCR-post-condensation reactions, especially for macrocycles, include intramolecular aryl couplings, amidations, S_n_Ar reactions, nucleophilic substitutions, and macrolactonizations. Even more interestingly, it is possible to perform the cyclization step via a second multicomponent reaction [22] or the MiB (*m*ultiple multicomponent macrocyclizations *i*ncluding *b*ifunctional building blocks) protocol developed by Wessjohann et al. (vide infra) [26].

## Review

### Small ring constraints

In the first part of this review four- to seven-membered cyclic peptidomimetics will be discussed. These small rings, particularly heterocycles, have received much attention as dipeptide mimics due to their capable interaction with defined protein motifs and due to their ease of preparation via IMCRs [27–29]. First, the β-lactams will be described followed by five-membered rings varying from pyrrolidines to tetrazoles based amide bond isosteres. Examples of the six-membered rings showing peptide like-properties are the piperazines, homoprolines, dihydropyrimidones and triazines, whereas azepines form an important class of seven-membered cyclic peptidomimetics.

#### Four membered ring constraints β-Lactams

**Scheme 6:**
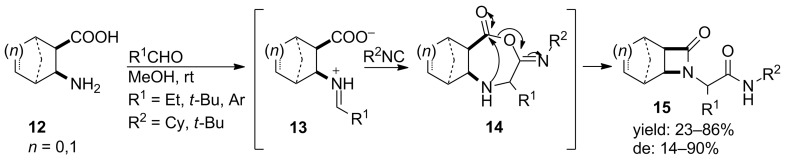
Mechanism of the U-4C-3CR towards bicyclic β-lactams.

The smallest class of cyclic peptidomimetics is that of the β-lactams. β-lactams are effective antibiotics [30] but also show inhibitory activities against serine- [31], elastase- [32–35], and HIV-1 protease [36] and papain [37]. For the design of β-lactams, the Staudinger reaction involving a [2 + 2] cycloaddition of ketenes and imines is the most common method used [38]. However, Ugi reactions starting form β-amino acids are also described. In 2002, the group of Fülöp reported an efficient synthesis of bicyclic β-lactams from monocyclic β-amino acids via an Ugi four-center three-component reaction (U-4C-3R) [39]. Herein, the monocyclic β-amino acid acts as bifunctional moiety containing both an amino and carboxylic acid group. A variety of cyclic β-amino acids, in which the ring was varied, were combined with a variety of aldehydes and isocyanides in methanol to obtain the desired β-lactams. In Scheme 6, a plausible mechanism of this reaction is shown.

The β-lactam ring herein is formed via a ring contraction of the seven-membered oxazepanone intermediate **14**, which in turn is formed from α-addition of the isocyanide to the bifunctional imine. Fülöp considered both racemic *cis-* and *trans-*β-amino acids, in which only the *cis*-racemates resulted in cyclized product **15**. The *cis*-products were obtained in moderate to good yields (23–86%) with diastereoselectivities varying from 14 to 90% (de). In 2007, they extended their protocol by performing the MCR in water, which is considered as environmentally benign, cheap and allowing simple isolation as the products precipitate [40]. Although, no improvements in yield or diastereoselectivity were observed, the reaction time was remarkably reduced in water (24 h vs. 72 h). One explanation for this acceleration could be the enhanced hydrogen bonding effect in the transition state [41]. Unfortunately, the construction of a large library was hampered, due to the poor solubility of several aldehydes in the aqueous media.

**Scheme 7:**
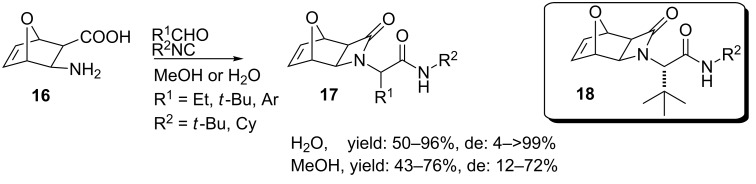
The Ugi 4C-3CR towards oxabicyclo β-lactams.

In a variation, the same group constructed a 10-membered library of oxabicyclo β-lactam derivatives (**17,** Scheme 7) from the bifunctional heteronorborene **16** in either water or methanol [42]. It was shown that both solvents gave similar results with regard to the yield (43–76% vs. 50–96%), whereas the diastereoselectivity was somewhat improved in water (12–72% vs. 4– >99%), in which the use of aliphatic aldehydes showed improved diastereoselectivity in this reaction. The highest diastereoselectivity was obtained with pivaldehyde **18** (100:0).

**Scheme 8:**
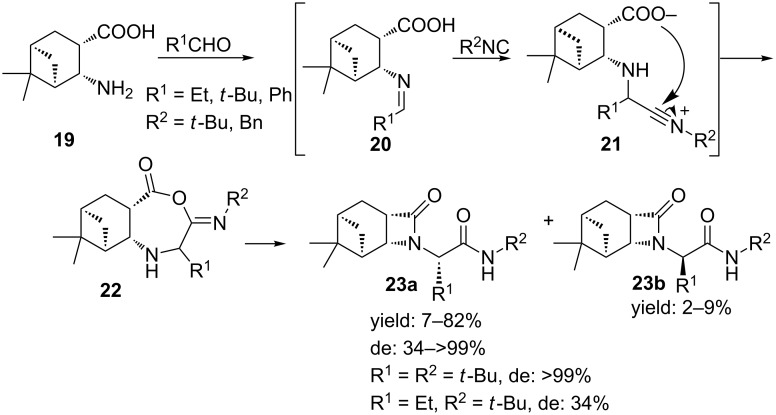
Ugi MCR between an enantiopure monoterpene based β-amino acid, aldehyde and isocyanide resulting in bicyclic β-lactams.

In 2010, Szakonyi et al. further extended their Ugi 4C-3CR-approach with enantiopure monoterpene-based β-amino acids [43] (**19**, Scheme 8), giving **22** as major isomer [44]. The stereoselectivity for **22** was explained by the steric effects of the dimethyl bridge that might prefer a *Re*-attack of the isocyanide. Compared to methanol, again the reaction in water proved to be faster. However, a solvent-free approach also resulted in the desired β-lactams with similar results in yield, diastereoselectivity and time.

**Scheme 9:**
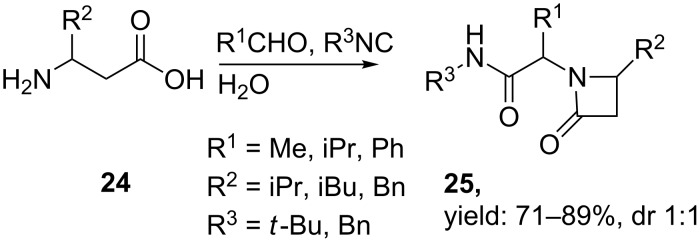
General MCR for β-lactams in water.

Besides bicyclic systems, Ugi 4C-3CRs towards monocyclic β-lactams are also described in both organic and aqueous media. Pirrung et al. published a library of 32 different β-lactams from four β-amino acids **24**, four different aldehydes and two isonitriles (Scheme 9) [41,45]. The reaction was performed in water at ambient temperature and yielded the desired products in good yields (71–89%), however, without diastereoselectivity (dr 1:1).

**Scheme 10:**
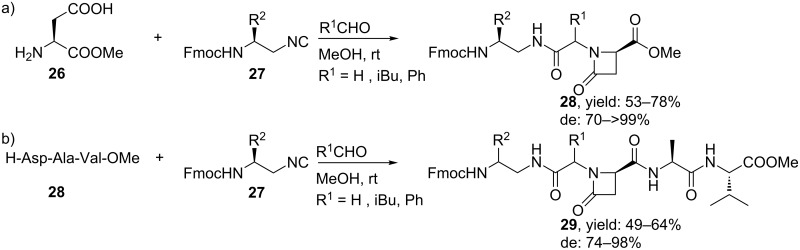
a) Ugi reaction for β-lactam-linked peptidomimetics. b) Varying the β-amino acid resulted in β-lactam-linked peptidomimetic structures.

To improve the diastereoselectivity, the group of Sureshbabu utilized chiral *N^β^*-Fmoc-amino alkyl isocyanides (obtained from *N^β^*-Fmoc-amino acids) and *L*-aspartic acid α-methyl esters as Ugi-substrates (Scheme 10) [46]. The resulting β-lactam-linked peptidomimetics were obtained in good yields (53–78%) with high diastereoselectivities (70–99%). In a variation, they also performed the reaction with *L*-aspartic acid α-peptide esters yielding *endo*-β-lactam mimics **29** in good yields (49–64%, de 74–98%).

#### Five-membered ring constraints

In natural peptides, the cyclic proteinogenic amino acid proline has stabilizing and turn-inducing properties determining the secondary and teriary structure and conformation of peptides [7,47–48]. Moreover, their influence on an altered *cis*/*trans* ratio of the amide bond has provided in-depth insights in conformation and receptor binding [49]. Thus, the specific properties of proline play a crucial role to determine the biological activity of peptides and peptidomimetics,[50] and research towards such peptidic structures containing proline-analogues has received much attention [48]. In this part, multicomponent reactions to access pyrrolidines and other five-membered derivatives such as γ-lactams, oxazoles, thiazoles and triazoles incorporated into peptide structures will be described.

#### Pyrrolidines

**Scheme 11:**
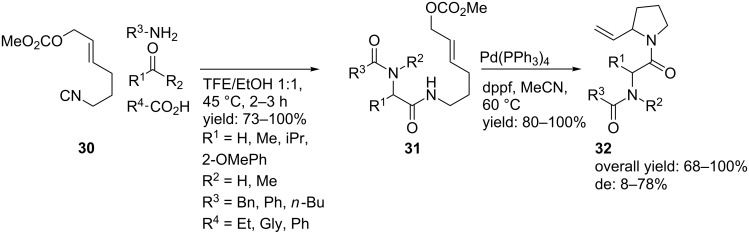
Ugi-4CR followed by a Pd-catalyzed S_n_2 cyclization.

2-substituted pyrrolidine-based dipeptide mimics were obtained from an Ugi-4CR followed by a Pd-catalyzed S_n_2 cyclization as described by Banfi et al. [51] . Herein, the Ugi reaction provided a small library of acyclic products (Scheme 11), in which the isocyanide input **30** was derived from the corresponding amine via an *N*-formylation/dehydration sequence [52]. An additional palladium-catalyzed cyclization gave the pyrrolidine mimics **32** in excellent yields and modest to good selectivities (de 8–78%). In addition, the mild conditions tolerate a wide range of Ugi-substrates, resulting in a broad range of different pyrrolidine mimics **32**.

**Scheme 12:**
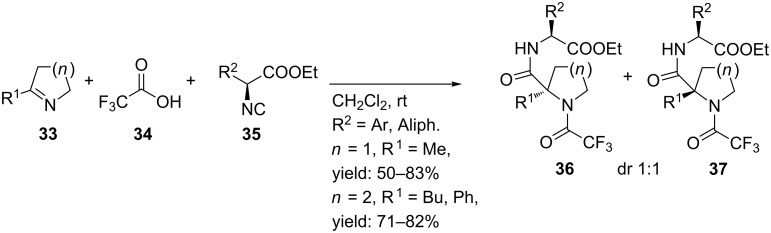
Ugi-3CR of dipeptide mimics from 2-substituted pyrrolines.

A more straightforward method [53] includes a single Joullié-Ugi 3CR using previously described alkyl substituted cyclic imines [54] giving the cyclic constraint peptides **36** and **37** (Scheme 12). In this work, no limitations regarding the type of isocyanide inputs were observed. Several alkyl-, aryl- and ester-substituted isocyanides gave the desired products. On the other hand, aryl-substituted pyrrolines as the imine input proved less efficient. The authors argued that the lower electrophilicity of arylimines and the possible enamide conjugation could account for this. Furthermore, it was shown that the pKa of the carboxylic acid significantly influenced the reaction rates, in which TFA gave the best results (2 days vs. 5 days for benzoic acid). No diastereoselectivity was observed in this reaction (dr 1:1) and the use of racemic isocyanides gave all four possible diastereomers*.*

**Scheme 13:**
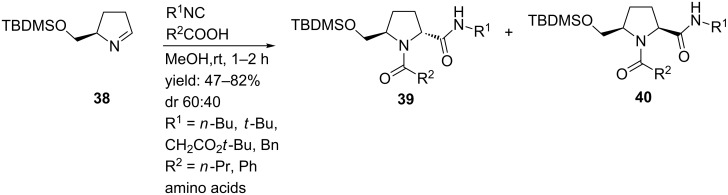
Joullié–Ugi reaction towards 2,5-disubstituted pyrrolidines.

**Scheme 14:**
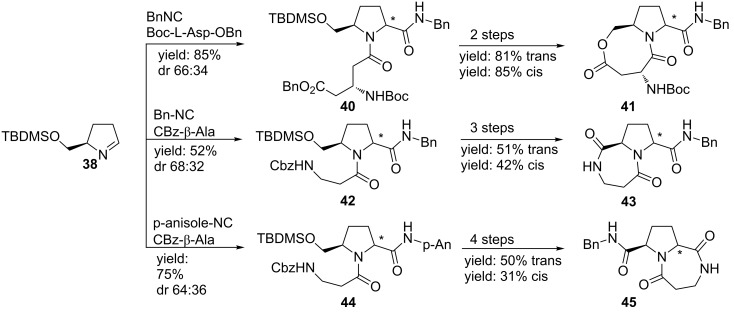
Further elaboration of the Ugi-scaffold towards bicyclic systems.

As an alternative, Banfi and co-workers focused on a library of 2,5-disubstitued pyrrolidines with potential external turn-motifs [55]. They described a Joullié–Ugi reaction from the highly reactive chiral pyrroline **38** and several carboxylic acids and isocyanides (Scheme 13). The disubstituted pyrrolidines were obtained in moderate to high yields, with a small preference for the *trans*-diastereomer **39** [56–57]. The judicious choice of carboxylic acid substituents allows a subsequent cyclization towards bicyclic systems (Scheme 14) such as pyrrolo-oxazepinediones **41** and pyrrolodiazepinediones **43**. The latter could be used as inhibitor for aminopeptidase P. In addition, incorporation of convertible isocyanides gave access to bicyclic compound **45** [13].

**Scheme 15:**
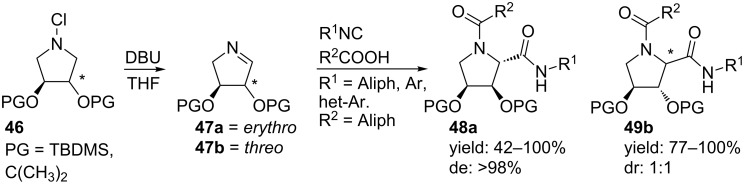
Dihydroxyproline derivatives from an Ugi reaction.

A one pot synthesis towards hydroxylated pyrrolidines was published by the group of Chapman (Scheme 15) [58]. Hydroxyproline derivatives have been reported as proline peptidase inhibitors [47]. The authors performed a Joullié–Ugi reaction with either the erythritol or the threitol imine **47a,b** and afforded both isomers **48a** and **49b**, respectively, in moderate to excellent yields. The reaction with the *erythro* isomer resulted in a single diastereomer **48a** whereas no selectivity was observed for the *threo* isomer.

**Scheme 16:**
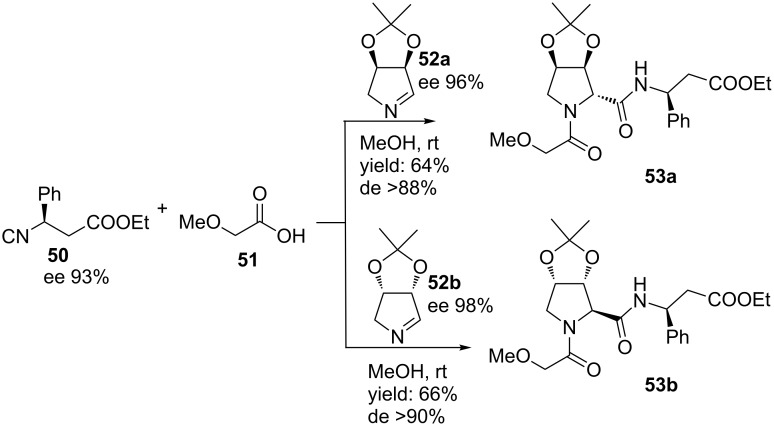
Diastereoselective Ugi reaction described by Banfi and co-workers.

**Scheme 17:**
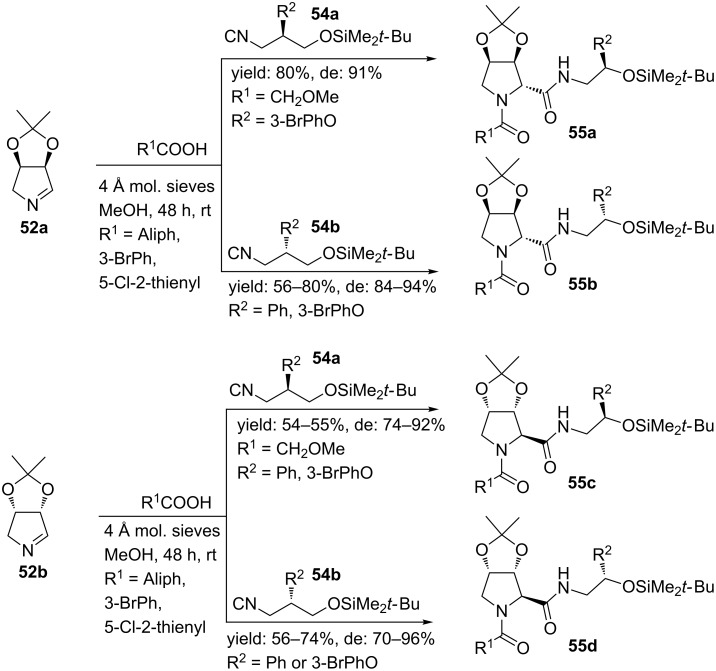
Similar Ugi reaction as in Scheme 16 but with different acids and two chiral isocyanides.

Based on this diastereoselective MCR, the group of Banfi developed an Ugi-Joullié 3-CR with carboxylic acids, chiral bicyclic imines and chiral isocyanides (Scheme 16) [59]. The chiral isocyanides were prepared following an organocatalytic phase-transfer Mannich-type reaction [59], whereas the chiral imines **52a**,**b** were obtained from a bio-catalytic protocol [60]. In particular, the rigid bicyclic imines are powerful starting points and they provide the Ugi-products **53a**,**b** in high yields and mainly as *trans-*isomers (de >88%), without racemization. As an extension, two other enantiopure isocyanides were combined with a variety of carboxylic acids furnishing a small library of bicyclic dipeptide mimics (**55a**–**d,** Scheme 17) in good yields and in high diastereomeric excess (de 70–96%) [60]. It is worth noting that deprotection of the acetal-group allows modulation of rigidity and polarity of the final molecules.

**Scheme 18:**
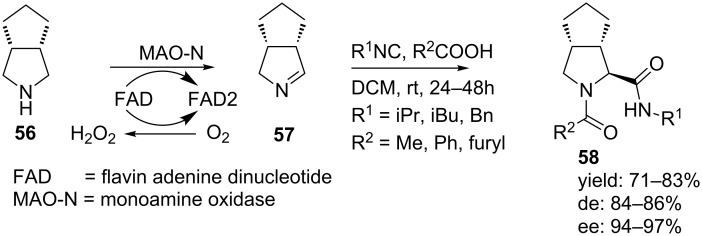
Highly diastereoselective synthesis of pyrrolidine-dipeptoids via a MAO-N/MCR-procedure.

Our group reported a highly diastereoselective Ugi-MCR towards 3,4-alkyl-substituted prolyl mimics by reacting several isocyanides and carboxylic acids with optically pure 3,4-*cis*-substituted imines **57** (ee 94–97%, Scheme 18) [61]. The chiral imines were derived from a biocatalytic oxidation of *meso*-pyrrolidines **56** using monoamine oxidase N (MAO-N) [62]. The Ugi-products were exclusively obtained as single *trans*-isomers in high yields (71–83%, de 84–86 % and ee 94–97%).

**Scheme 19:**
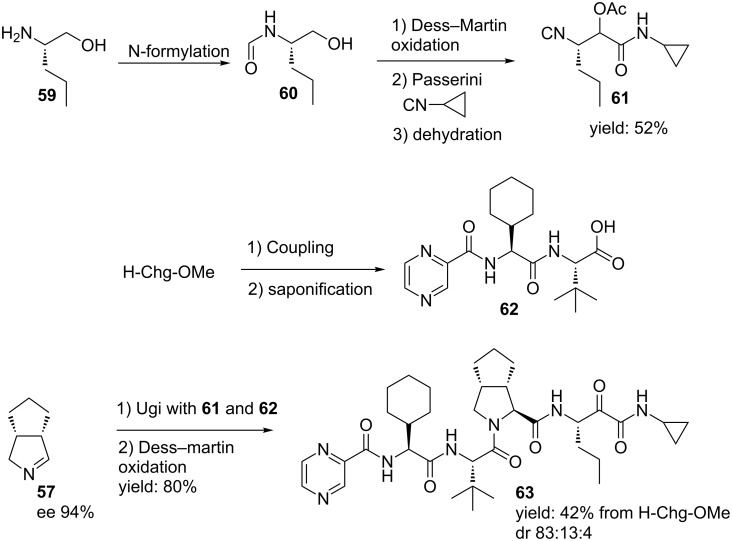
MAO-N/MCR-approach towards the hepatitis C drug telaprevir.

It is noteworthy that these enantiopure imines can be used in a MCR-based approach to access telaprevir, a known protease inhibitor of hepatitis C (Scheme 19) [63]. The key steps in this route are a Passerini 3-CR to afford the isocyanide substrate **61** and a subsequent Ugi 3-CR/oxidation protocoll to provide the final compound in a much shorter and more straightforward route compared to earlier described syntheses (11 vs. 24 steps). The bicyclic imine **57** was obtained via the MAO-N desymmetrizaton described above, whereas a peptide-coupling between L-cyclohexylglycine methyl ester pyrazinecarboxylic acid followed by a saponification afforded the carboxylic acid **62**. Moreover, for the isocyanide component, the Dess–Martin oxidation of **60** and the subsequent Passerini reaction could be performed in one-pot, since the former reaction produces acetic acid as byproduct. Addition of cyclopropyl isocyanide followed by dehydration of the Passerini-product furnished the third Ugi-component **61**. The subsequent Ugi 3-CR of **61**, **62** and **57** followed by a final oxidation resulted in **63** (42% from H-Chg-OMe, dr 84:13:4).

**Scheme 20:**
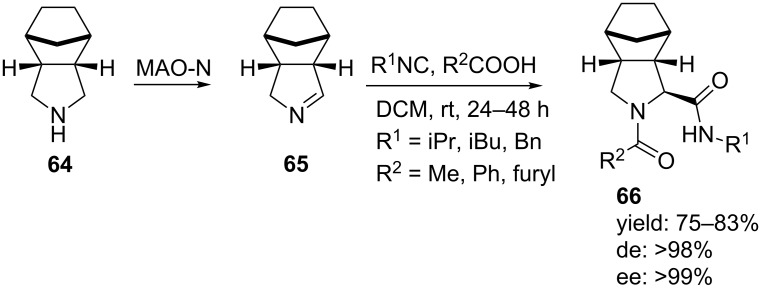
Enantioselective MAO-U-3CR procedure starting from chiral pyrroline **64**.

Even better selectivities were observed using the more sterically hindred **64** (Scheme 20) [61] in a similar MAO-N/MCR combination. In this way, dipeptide mimics **66** were obtained in good yields (75–83%), however, now with very high diastereomeric (>98%) and enantiomeric excesses (>99%).

#### γ-lactams

**Scheme 21:**
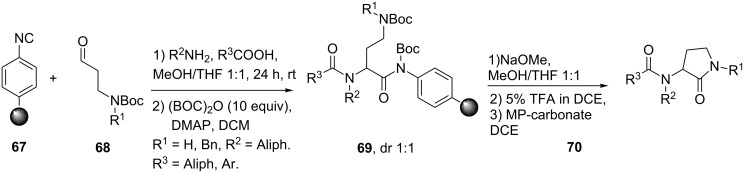
Synthesis of γ-lactams via an UDC-sequence.

**Scheme 22:**
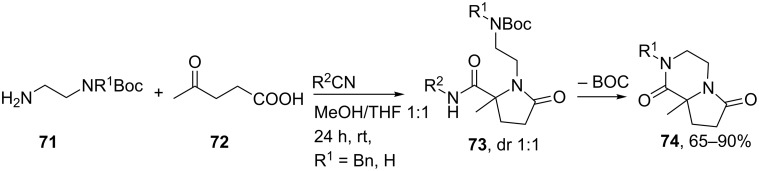
Utilizing bifunctional groups to provide bicyclic γ-lactam-ketopiperazines.

The γ-lactam unit is an important dipeptide pharmacophore since it can induce β-turns. A well-known example was described by Freidinger in 1980, who successfully developed a γ-lactam β-turn mimic of the luteinizing hormone-releasing hormone (LHRH) almost nine times more potent than the original hormone [64]. Since then, these “Freidinger lactams” have been used in numerous pharmaceutical and biological active compounds. For example, they are found in compounds used for the treatment of epilepsy [65–66], HIV [67–68], and depression [69]. Multicomponent reactions towards γ-lactam peptidomimetics were earlier described by Ugi [70], Mjalli [71] and Harriman [72]. However, in the last decade two other groups, independently, published Ugi-MCRs towards these cyclic dipeptide isosteres. Hulme et al. reported an Ugi-Deprotection–Cyclization strategy using resin-bound convertible isonitrile **67** to provide primary and secondary γ-lactams **70** in high purities over five steps (Scheme 21) [73]. As an extension, they also performed the reaction sequence with bifunctional building blocks **71** and **72**, in which subsequent *N*-Boc-deprotection of **72** provided bicyclic γ-lactam-ketopiperazines **74** (Scheme 22).

**Scheme 23:**
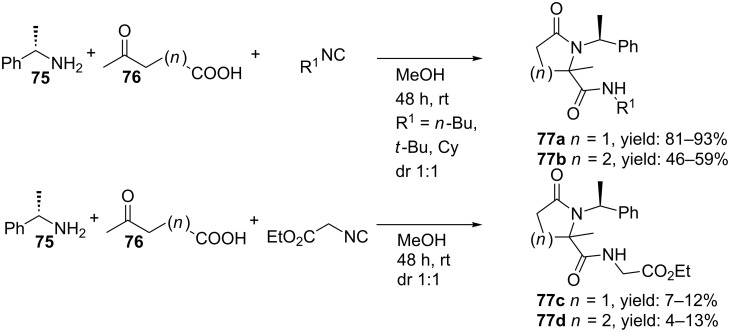
The Ugi reaction provided both γ- as δ-lactams depending on which inputs were used.

**Scheme 24:**
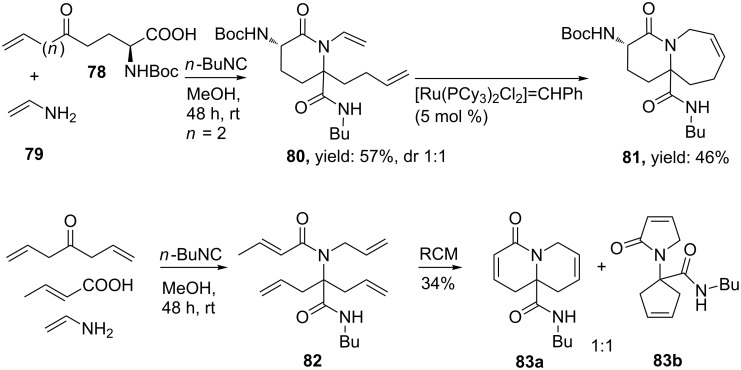
The sequential Ugi/RCM with olefinic substrates provided bicyclic lactams.

Krelaus et al. described the one-pot synthesis of both γ- and δ-lactams from an Ugi 4C-3CR (Scheme 23) [74]. Screening a variety of isocyanides resulted in a small library of γ-lactams **77a** and δ-lactams **77b**, in which the former were obtained in higher yields probably due to a more favourable six-membered transition state in the Mumm-rearrangement. Moreover, the nucleophilicity of the isocyanide used also seems important. Thus, isocyano butane provided the γ-lactams in high yields (81–93%), whereas more acidic ethyl 2-isocyano acetate showed less efficient conversion (7–13%). The stereoselectivity of the process was also studied, however, even with two chiral inputs no stereoinduction was observed for the newly formed stereocenter (dr 1:1). As an extension, the authors performed the Ugi reaction with allyl amine **79** and olefinic amino acids **78** (derived from pyroglutamic acid), that, after a following ring-closure-metathesis (RCM) with Grubb’s catalyst, resulted in bicyclic lactams (**81**, 46% over the two steps, Scheme 24). In addition, an even shorter route by utilizing three olefinic Ugi-substrates was also reported. Herein, the ring closing step included a double RCM and resulted in an equal amount of both products **83a** and **83b** (ratio 1:1) [75–76].

#### Triazoles

**Scheme 25:**
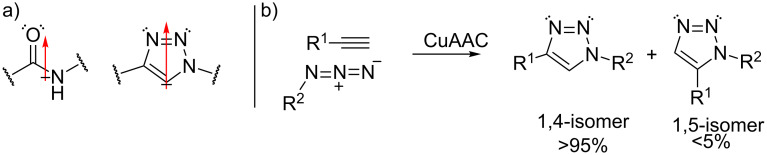
a) The structural and dipole similarities of the triazole unit with the amide bond. b) The copper-catalyzed Click reaction.

The replacement of amide bonds by 1,2,3-triazoles, especially the 1,4-disubstituted isomer, provided a wide variety of biological active peptidomimetics. Peptidomimetics containing these triazole cores can serve as blood components [77], anticancer medications [78], inhibitors of cysteine [79] and HIV-1 proteases [80–82]. The relative planarity of 1,2,3-triazoles, the strong dipole moment (~5 D) and the ability to both donate and accept hydrogen bonds indicate the physicochemical similarities with amide bonds (Scheme 25), however, they are inert towards oxidation, hydrolysis and enzymatic degradation [88]. Several studies have revealed the bio-similarity of triazoles with amide bonds. For example, X-ray studies towards triazole based-mimics of the HIV-1 protease inhibitor amprenavir showed an equivalent binding mode with the protease active site as compared to the amide-bond inhibitor [81,83–84].

Multicomponent reactions towards amide isosteres often involve an Ugi reaction followed by a Click reaction, in which two of the Ugi-inputs either contain an alkyne or an azide moiety. A well-known example of the latter reaction is the Copper(I) catalyzed azide–alkyne cycloaddition (CuAAC) between acetylenes and azides (Scheme 25) [85]. Advantages of this reaction are the kinetic stability of both functional groups under a range of different conditions. Also, the triazole products can be formed in both organic and aqueous solvents and by using the Cu(I)-catalyst which induces regioselective formation of the 1,4-isomer over the 1,5-isomer [85].

**Scheme 26:**
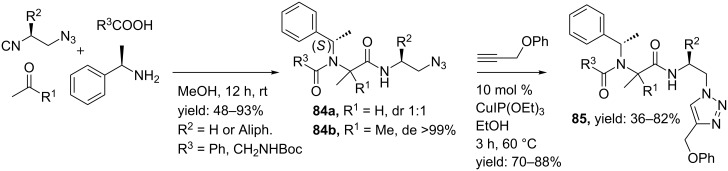
The Ugi/Click sequence provided triazole based peptidomimetics.

In 2010, Nenajdenko et al. described an Ugi/Click-approach using chiral isocyanoazides, which in turn were derived from L-amino alcohols [85]. The Ugi-products were obtained in good yields, with high diastereoselectivity (de >99%). A follow-up Click reaction using 10 mol % CuIP(OEt)_3_ and phenyl propargyl ether as the alkyne provided triazole-peptidomimetics **85** in 70–80% yield (Scheme 26).

**Scheme 27:**
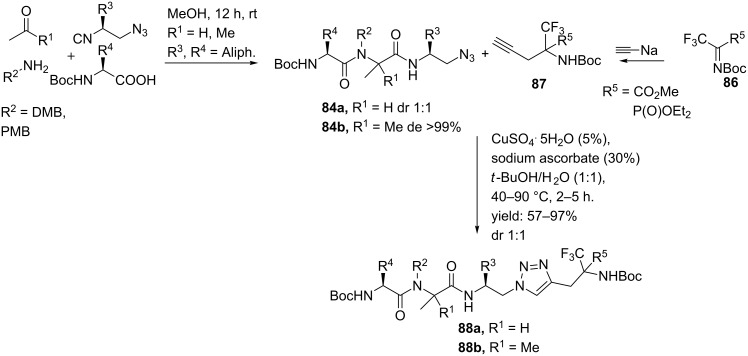
The Ugi/Click reaction as described by Nanajdenko.

In a follow-up publication the same authors reported the development of tetrapeptides bearing α-CF_3_-α-amino and α-CF_3_-α-amino phosphonate cores (Scheme 27) [86]. Fluorinated compounds may enhance the biological properties of target molecules and especially CF_3_-containing amino acids have demonstrated to be hydrolytically more stable as compared to the native amino acids [87]. In addition, the insertion of α-amino phosphonates to peptides has shown enhanced antibacterial, antivirus and anticancer activities [88]. The Ugi reaction provided the azide moieties **84a** or **84b**, whereas a reaction between sodium acetylide and imines of general structure **86** afforded the alkyne derivatives [89–90]. A subsequent Click reaction gave the final triazole-mimics (**88a** or **88b**) in good to excellent yields (57–97%).

**Scheme 28:**
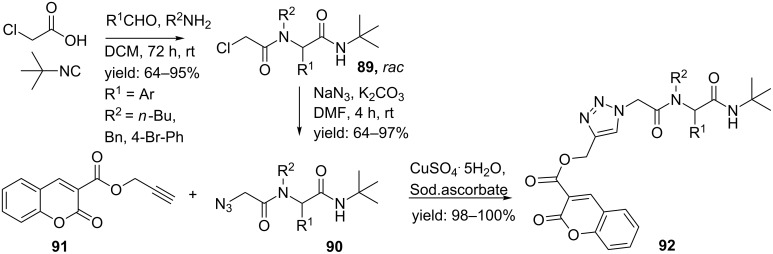
The Ugi/Click-approach by Pramitha and Bahulayan.

A less common approach was reported by Pramitha and Bahulayan [91]. Herein, the Ugi reaction was performed with chloroacetic acid, *tert*-butyl isocyanide and different aldehydes and amines yielding chloro-Ugi products **89** (Scheme 28). A subsequent substitution with sodium azide followed by the Click reaction resulted in the triazole-linked peptidomimetics **92**.

**Scheme 29:**
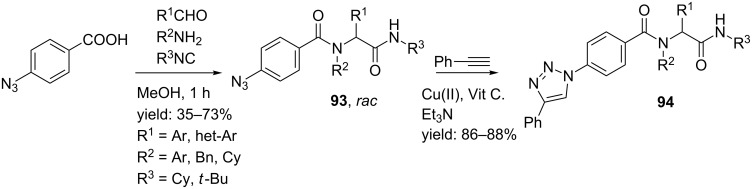
The Ugi/Click-combination by Niu et al.

Recently, Niu et al. reported an Ugi/Click method to obtain peptidomimetics that have triazole units at the terminal, center and/or at the side chain position [92]. Terminal triazoles were obtained via an Ugi reaction of 4-azidobenzoic acid and different isocyanides, aldehydes and amines (Scheme 29). No limitations for the amine substrate were observed, whereas electron-withdrawing groups in the aldehyde decrease the yield compared to electron donating groups. For practical reasons, the authors used Cu(OAc)_2_/vitamin C/Et_3_N as Cu^II^-complex, in which vitamin C functioned as reducing agent.

**Scheme 30:**
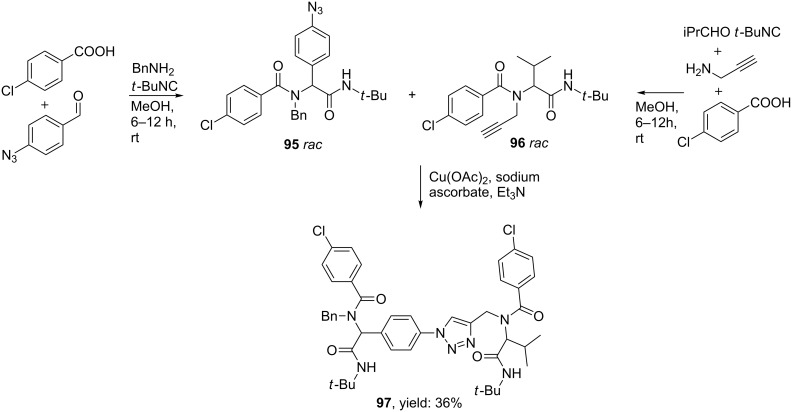
Triazole linked peptidomimetics obtained from two separate MCRs and a sequential Click reaction.

Side-chain triazoles were obtained from 4-azidobenzaldehyde in 48–62% yield, whereas incorporation of both 4-azidobenzaldehyde and 4-azidobenzoic acid provided mimics containing both a triazole unit in the side chain as in the terminal part (43%). More interestingly, the authors also described an Ugi/Click-combination in which both Click-substrates were obtained from an Ugi reaction. The subsequent Click cycloaddition provided the triazole linker **97** in 36% overall yield (Scheme 30). The authors also considered the possibility to perform these three steps simultaneously in one-pot, however, as isocyanides are also good ligands to transition metals, the Ugi products were only obtained in low yield (19%) without observing any triazole product.

**Scheme 31:**
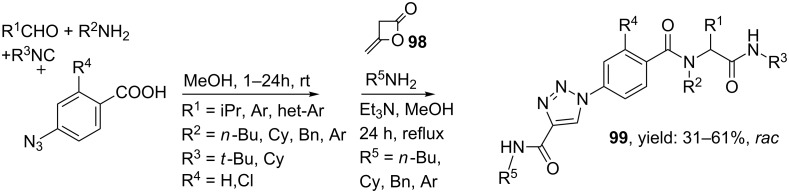
Copper-free synthesis of triazoles via two MCRs in one-pot.

Even more interestingly was the copper-free procedure published by the group of Cai, especially since copper was found in some cases to be toxic to bacterial and mammalian cells [93]. Through a sequential Ugi 4-CR and a three-component cycloaddition they exclusively prepared 1,4-disubstituted triazoles (**99**, Scheme 31). Thus, the Ugi 4-CR produced the azide precursor that further reacted in the subsequent 3-CR with diketene **98** and a wide variety of primary amines to afford the triazole linker. The scope of the Ugi reaction was investigated with several aliphatic and aromatic aldehydes and amines and gave the Ugi-products in good yields (34-61%). Moreover, the scope of sequential cycloaddition was also explored with several aliphatic and aromatic amines, in which the more electron-rich inputs gave the higher yields. In a variation, first the triazole-unit was formed followed by the Ugi MCR. However this resulted in lower yields compared to the initial sequence (33–39% vs. 31–61%).

#### Pyrazoles and pyrazolones

**Scheme 32:**
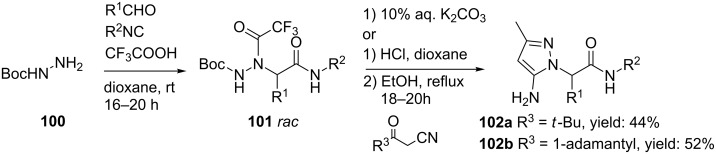
The sequential Ugi/Paal–Knorr reaction to afford pyrazoles.

**Scheme 33:**
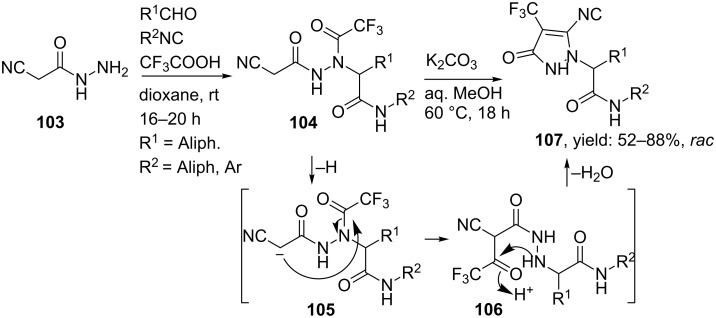
An intramolecular Paal–Knorr condensation provided under basic conditions pyrazolones.

**Scheme 34:**
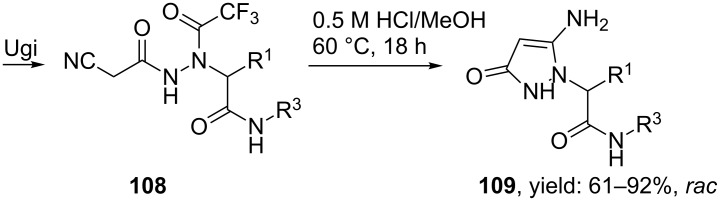
Similar cyclization performed under acidic conditions provided pyrazolones without the trifluoroacetyl group.

The group of Krasavin prepared both pyrazole- as well as pyrazolone-containing peptidomimetics via a sequential hydrazino-Ugi/Paal–Knorr condensation [94]. In their first approach, pyrazoles were obtained in good yields, however, with a limited scope of the condensation substrates (Scheme 32). Therefore, an intramolecular Paal–Knorr condensation of **104,** derived from an Ugi reaction of bifunctional hydrazine **103** (Scheme 33), under basic conditions was considered. Surprisingly, use of a base did not cleave the trifluoroacetyl group but instead deprotonation of the methylene group was found, yielding pyrrazol-3-ones **107**. The authors assumed that the cyclization proceeds via an N–C acyl migration of the trifluoroacetyl group, based on the labile character of the latter moiety (Scheme 33). In contrast, acid-promoted cyclization cleaved the trifluoroacetyl group and revealed the initially expected compound **109** (Scheme 34).

#### Thiazoles

**Scheme 35:**
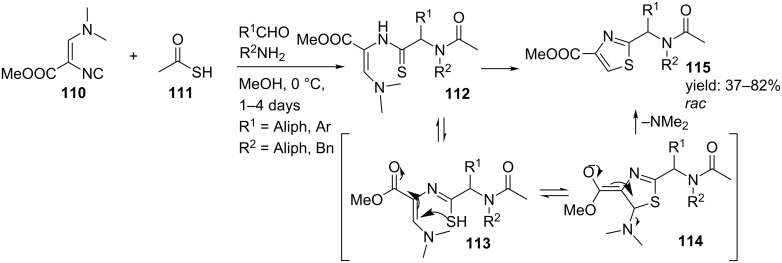
The Ugi-4CR towards 2,4-disubstituted thiazoles.

In several natural products, the thiazole ring can be found as a backbone linker, probably resulting from easy cyclization/oxidation of cysteine residues. These compounds show interesting antifungal and antibiotic [95–96], algicidal [97], and antitumor [98–99] biological activities. In addition, thiazole-based pharmaceuticals are also used as anti-prion agents (neurodegenerative disorders) [100]. Dömling and co-workers were the first that reported an Ugi-based synthesis of 2,4-disubstituted thiazoles (Scheme 35) [101–102]. The procedure involved a condensation of isocyanoacrylate **110** (derived from a known protocol by Schöllkopf) [103], thiocarboxylic acid, a variety of aliphatic amines and several aliphatic and aromatic oxo-components, furnishing the thiazoles **115** as racemic mixtures in moderate to excellent yields (37–82%). Based on the fact that the Ugi-product tautomerizes, the authors proposed a plausible mechanism, in which tautomer **113** undergoes cyclization via an intramolecular Michael reaction to give intermediate **114**. The next step involves cleavage of the dimethylamine group to afford the thiazole structures **115**.

**Scheme 36:**
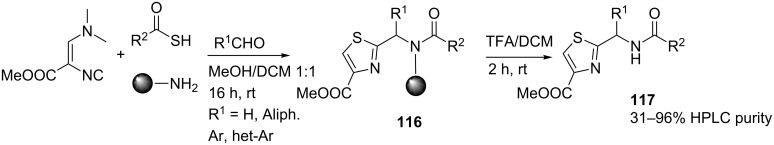
Solid phase approach towards thiazoles.

In 2003, the same group also described a solid-phase approach with either thioacetic acid or thiobenzoic acid to obtain the 2-acylaminomethylthiazoles [104]. Deprotection of the resin onto the amide, resulted in compound **117** (Scheme 36).

**Scheme 37:**
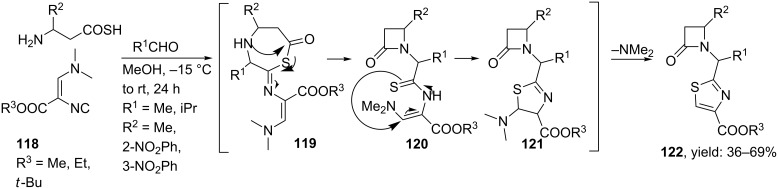
Reaction mechanism of formation of thiazole peptidomimetics containing an additional β-lactam moiety.

As an extension, Dömling and co-workers designed thiazole-based dipeptide mimics with an additional β-lactam moiety attached to the scaffold. These compounds could find application as potent antibiotics, protease inhibitors or as cholesterol absorption modifiers (Scheme 37) [18]. In this particular reaction, the scaffolds were obtained by reacting the complex isocyanide **118** with different aldehydes and β-amino thiocarboxylic acids, in which the latter component was obtained from Z-protected β-amino acids via a cyclic anhydride in two steps [105]. Most likely this reaction proceeds via a 7-membered Ugi-intermediate that after an intramolecular acylation results in β-lactam intermediate **120**. A subsequent Michael-type addition followed by dimethylamine absorption then affords the observed thiazoles (((1-thiazole-2-yl)methyl)azetidin-2-ones) in moderate to good yields (36–69%).

**Scheme 38:**
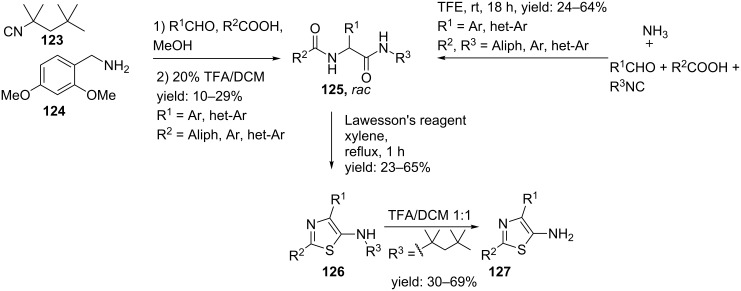
The synthesis of the trisubstituted thiazoles could be either performed via an Ugi reaction with protected amines or with ammonia.

In contrast, Thompson et al. described the synthesis of 2,4-disubstituted 5-aminothiazoles via a sequential Ugi/deprotection/thionation/cyclization strategy, in which both R^1^ and R^2^-positions could be easily varied (Scheme 38) [106–107]. They derived the linear dipeptide from an Ugi 4-CR involving the Walborsky reagent **123** (1,1,3,3-tetramethylbutyl isocyanide) as a cleavable isocyanide input, 2,4-dimethoxybenzylamine **124** (DMB–NH_2_), different aldehydes and carboxylic acids [106]. Subsequent TFA-treatment provided the precursor **125** that via a follow-up reaction with Lawesson’s reagent and an intramolecular cyclization gave access to the thiazole derivative **126**. A second TFA-cleavage of the *N*-(1,1,3,3-tetramethylbutyl) group resulted in the 5-aminothiazole peptidomimetics **127** in sufficient overall yields (5–13%).

**Scheme 39:**
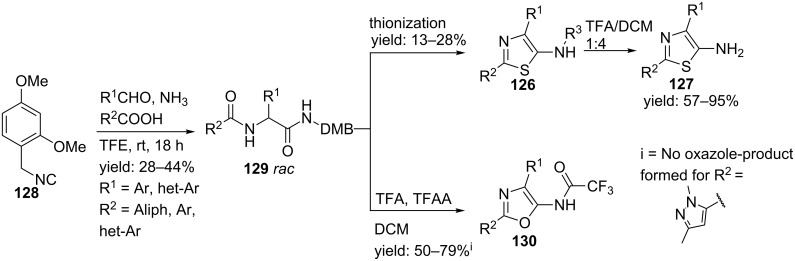
Performing the Ugi reaction with DMB-protected isocyanide gave access to either oxazoles or thiazoles.

In a variation, the authors designed an ammonia-based Ugi reaction that avoids the use of protected amines (Scheme 38) [107]. It was shown that this protecting-group-free protocol tolerates a great variety of different isocyanides and also allows acid sensitive substrates. In particular, the use of isocyanide **128** gave access to 5-aminothiazoles **127** after deprotection of the DMB-group (Scheme 39).

**Scheme 40:**
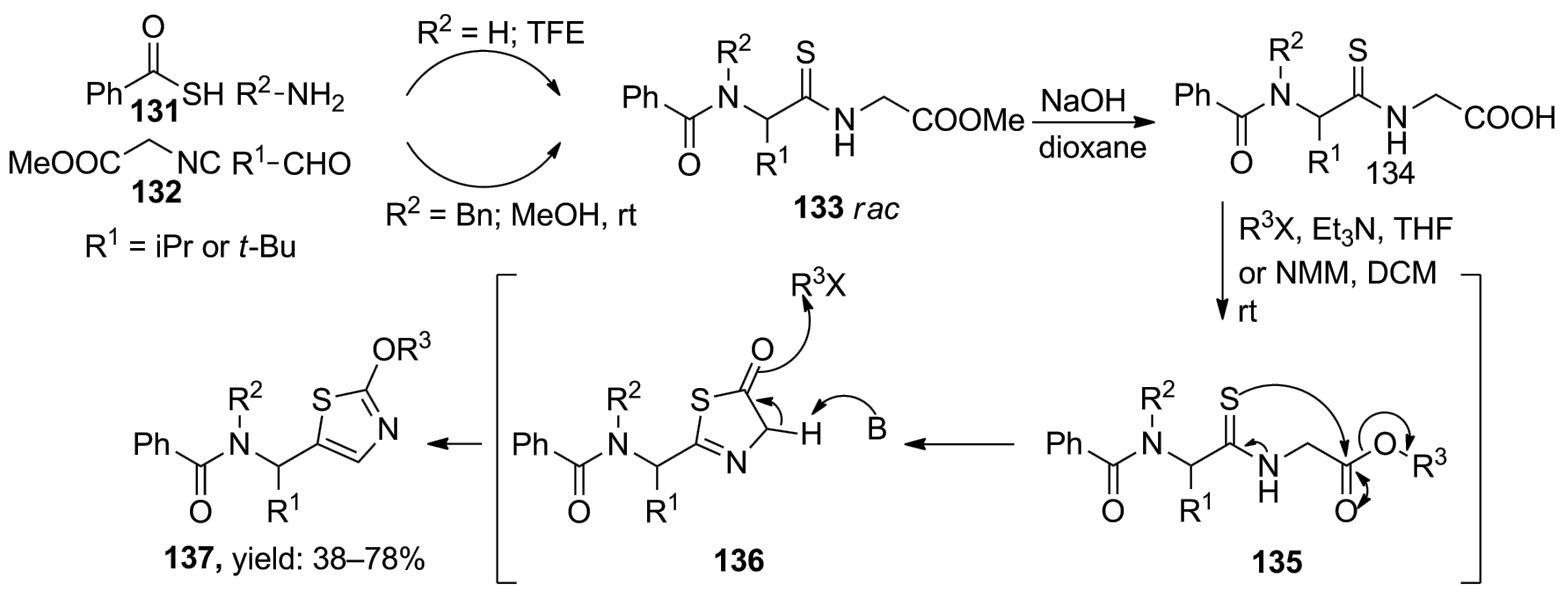
Ugi/cyclization-approach towards 2,5-disubstituted thiazoles. The Ugi reaction was performed with different benzylamines or ammonia.

**Scheme 41:**
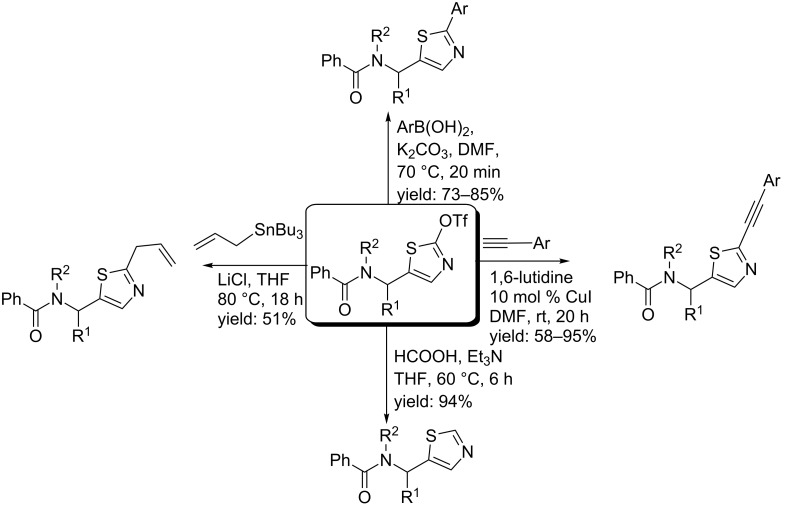
Further derivatization of the thiazole scaffold.

Kazmaier and Persch studied a versatile thio-Ugi MCR towards 2,5-disubstituted thiazoles (Scheme 40). The authors first obtained the Ugi-products by reacting thiobenzoic acid, isocyanoacetate, two aliphatic aldehydes (iPr, *t*-Bu) and benzylamines/ammonia in either methanol (for benzylamines) or trifluoroethanol (for ammonia) [108]. Then, subsequent hydrolysis of the methylester followed by activation via acid chlorides or triflates gave the 5-substituted thiazoles (38–78%), probably via a thiazolone intermediate. It is noteworthy that the triflate-thiazoles can be further elongated via cross coupling reactions, resulting in even higher functionalized 5-substituted or non-functionalized thiazoles in good to excellent yields (51–95%, Scheme 41) [108].

**Scheme 42:**
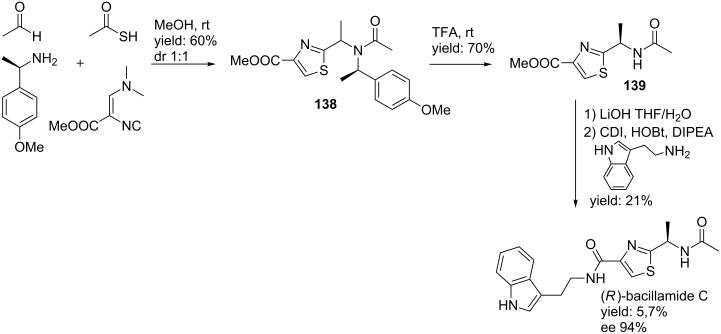
Three-step procedure towards the natural product bacillamide C.

In 2009, the group of Dömling reported an Ugi-approach towards the synthesis of Bacillamide C (Scheme 42) [109]. (*R*)-Bacillamide C is a natural product with algicidal and antibacterial properties [97]. It was shown that a stereoselective reaction between Schöllkopf’s isocyanide, acetaldehyde, thioacetic acid and 4-methoxy-phenylethylamine (also as chiral auxiliary) provided the corresponding Ugi product **138** in 60% yield (dr 1:1). Chiral separation and deprotection in TFA resulted in compound **139** in 70% yield, after which saponification followed by an amide coupling with tryptamine and CDI afforded the final (*R*)-bacillamide C in 6% yield over three steps (ee 94%).

#### Oxazoles

**Scheme 43:**
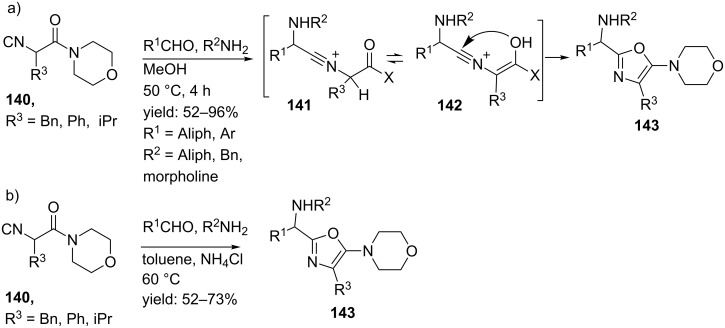
Ugi-4CR to oxazoles reported by Zhu and co-workers.

The oxazole unit has been applied in different bioactive marine natural products [110]. The group of Zhu reported a Ugi 3-CR to a small library of 2,4,5-trisubstituted oxazole-containing peptide-like structures from bifunctional α-isocyanoacetamides (Scheme 43) [111–112]. A plausible mechanism for this reaction involves the formation of **141**, that after tautomerization, cyclizes to the oxazole product **143**. It is noteworthy, that this reaction proceeds without the addition of a carboxylic acid because amino-oxazoles are unstable under acidic conditions. In total, six different aldehydes, twelve amines and three isocyanides yielded the corresponding desired oxazole mimics **143** in good yields (60–96%), however, the products were obtained as racemates even when the reaction was performed with chiral isocyanides. Some diastereoselectivity was only observed when the methyl ester of (*S*)-proline was used as an amine input (de 42%, Scheme 43). As an extension, the authors also performed the Ugi reaction in (the non-polar solvent) toluene and ammonium chloride as proton source (for the imine) at evaluated temperatures to provide the oxazoles in 52–73% yield (Scheme 43). It is noteworthy that during this latter approach no Passerini products or side-products with NH_3_ (from ammonium chloride) were observed.

**Scheme 44:**
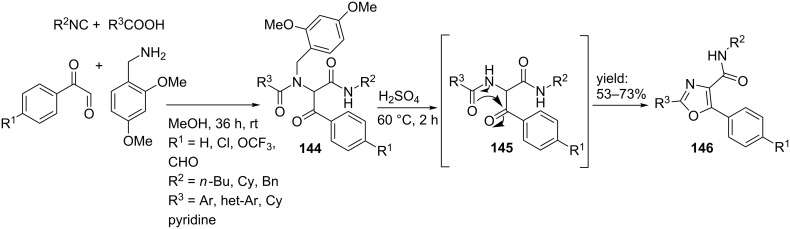
Ugi-based synthesis of oxazole-containing peptidomimetics.

In contrast, Shaw et al. [113] reported another convenient approach in which a sequential Ugi 4-CR followed by a Robinson–Gabriel reaction resulted in the desired oxazoles (Scheme 44). Herein, dimethoxybenzylamine, several isocyanides, aryl-glyoxals and (hetero)-aryl carboxylic acids afforded the desired corresponding Ugi-products **144** in reasonable to good yields (42–65%). Subsequent exposure to concentrated sulfuric acid at 60 °C deprotected and cyclized the linear products towards the oxazole derivatives **146** in yields up to 73%.

#### Tetrazoles

In medicinal chemistry, tetrazoles are often used as carboxylic acid bio-isosteres due to their comparable acidity [114]. However, studies towards 1,5-disubstituted tetrazoles have shown that these heterocycles also show geometrical properties similar to *cis*-amide bonds [115]. Therefore, they have been incorporated as constrained *cis*-amide bond isosteres in several bio-active compounds such as inhibitors of cyclooxygenase-2 (COX-2) [116], hepatitis C NS3 proteases [117], HIV-1 proteases [111–112,117], the CB1 receptor of cannabinoid and fatty acid amide hydrolase [114,118–121].

**Scheme 45:**
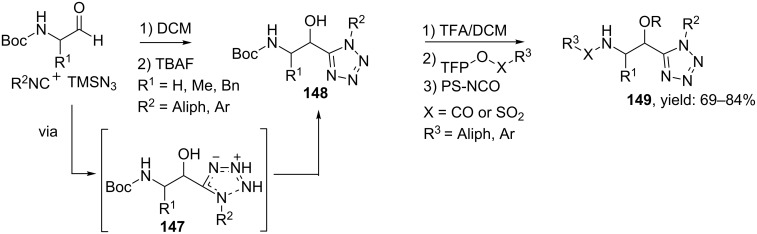
TMNS_3_ based Ugi reaction for peptidomimics containing a tetrazole.

In this context, Hulme and co-workers described a Passerini multicomponent approach towards *cis*-constrained norstatine mimics, a class of HIV-1 protease inhibitors with a tetrazole core (Scheme 45) [122]. They showed that a TMSN_3_-modified Passerini 3-CR gave easy access to tetrazole building blocks that, after *N*-Boc-deprotection, could be coupled with polymer-bound tetrafluorophenol-esters. Subsequent heating provided the desired *N*-coupled Norstatine peptidomimetics **149** (HPLC purities: 30–74%), in which additional scavenging of the unreacted amines with polystyrene-based isocyanate (PS-NCO) improved the purities of the final products (69–84%). It is noteworthy that the use of TMSN_3_ has several advantages, as it is less toxic and explosive than commercial derivatives and the byproduct (methoxytrimethylsilane) is easily evaporated.

**Scheme 46:**
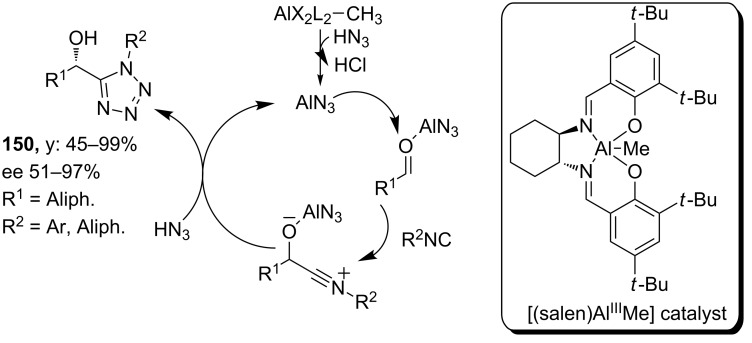
Catalytic cycle of the enantioselective Passerini reaction towards tetrazole-based peptidomimetics.

Based on this Passerini reaction, the group of Zhu developed an enantioselective approach using hydrazoic acid as the azide source and [(salen)Al^III^Me] as the catalyst [123]. A variety of aliphatic aldehydes and both aliphatic and aromatic isocyanides were tolerated in their approach and resulted in a library of tetrazoles **150** with excellent yields and high enantiomeric excesses (51–97%). For this enantioselectivity, the authors proposed a mechanism as shown in Scheme 46. In addition, from their study it became clear that the azide moiety is directly transferred from HN_3_ and not from the Al-bound azide, since no product was formed in the absence of HN_3_.

**Scheme 47:**
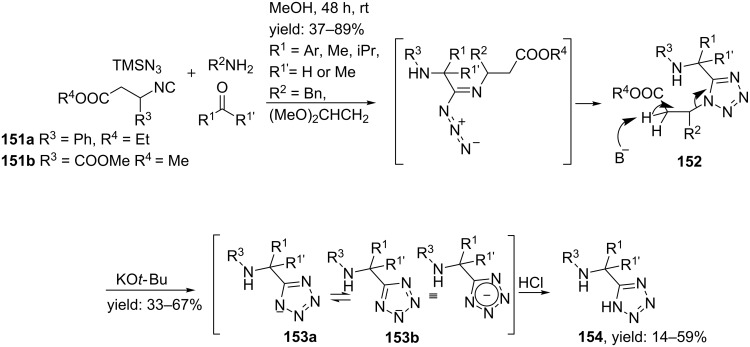
Tetrazole-based peptidomimetics via an Ugi reaction and a subsequent sigmatropic rearrangement.

5-substituted tetrazoles could also be obtained from an Ugi 4-CR between aldehydes, amines and TMSN_3_ and cleavable isocyanides as was described by Mayer et al. [124] Cleavable isocyanides consist of acidic protons at the β-position and can be obtained for example from β-amino acids. During the Ugi reaction, the tetrazole moiety is obtained from a sigmatropic rearrangement (Scheme 47). Subsequent base-treatment enables β-elimination, which is driven by mesomeric stabilization of the triazole ring, resulting in the desired 5-substituted tetrazoles **154** in moderate to good yields with three points of diversity.

**Scheme 48:**
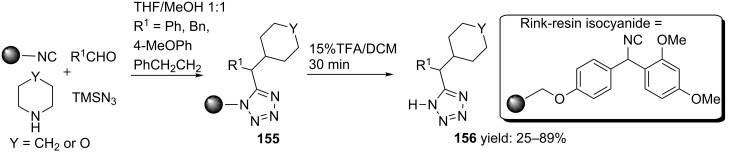
Resin-bound Ugi-approach towards tetrazole-based peptidomimetics.

Alternatively, 5-substituted tetrazoles can be obtained via an Ugi–reaction between a (Rink) resin bound isocyanide, TMSN_3_, several aldehydes and amines [125]. The final tetrazoles **156** were obtained from TFA cleavage (Scheme 48).

**Scheme 49:**
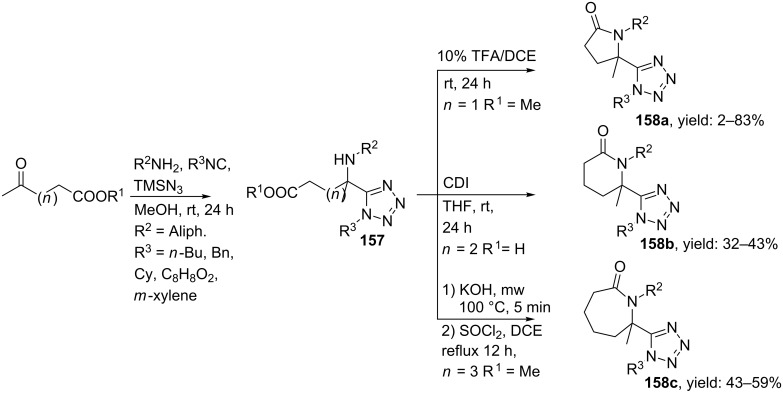
Ugi/cyclization approach towards γ/δ/ε-lactam tetrazoles.

In a combined approach, Gunawan et al. also described such an Ugi-azide reaction to develop γ/δ/ε-lactam tetrazoles [126–127]. Depending on the keto-ester or acid used, an Ugi reaction with different primary amines, isocyanides and TMSN_3_ followed by subsequent cyclization gave either γ-, δ- or ε-lactam tetrazoles (Scheme 49). Herein, cyclization for the γ-lactam derivatives was performed under acidic conditions, while CDI was used as cyclization agent in the δ-lactam formation and SOCl_2_ was required for the ε-lactam tetrazoles. All multicomponent reactions were performed in MeOH at room temperature and the final tetrazoles **158a–c** were obtained in moderate to good yields.

#### Six-membered ring constraints

**Pipecolic acid**

**Scheme 50:**
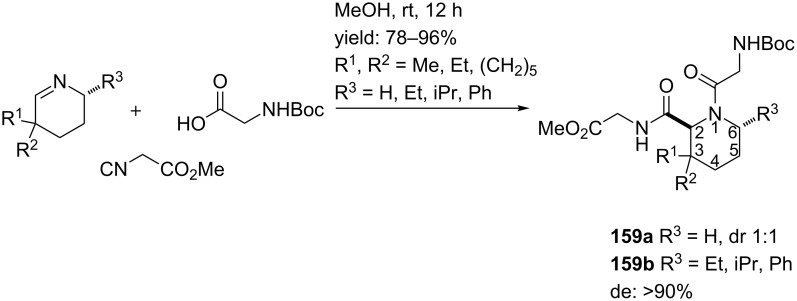
Ugi-3CR to pipecolic acid-based peptidomimetics.

In the previous section we discussed some important conformational properties of proline derivatives playing an important role in controlling peptide and protein secondary structures. Replacement of the proline residue by its six-membered analogue, pipecolic acid, has provided valuable insights in peptide folding and bioactive conformations [128–129]. In particular, pipecolic acid derivatives often find their application as β-turn mimetics [130], and are therefore included in several pharmaceutical compounds such as antipsychotics, anticonvulsants, local anaesthetics or analgesics [131]. An interesting diastereoselective multicomponent approach towards such six-membered pipecolic acid-based analogues was described by Maison et al. [128] Although this work is closely related to earlier work of Dömling and Ugi [129], it is an interesting extension of the original protocol. Maison investigated 3- and 6-substituted pipecolic acid analogues **159a–b** via a reaction with achiral and chiral imines, methyl-2-isocyanoacetate and *N*-Boc-protected glycine (Scheme 50). It was shown that the products were obtained in excellent yields and in high diastereoselectivity when chiral imines were employed (**159b**, de >95%).

**Scheme 51:**
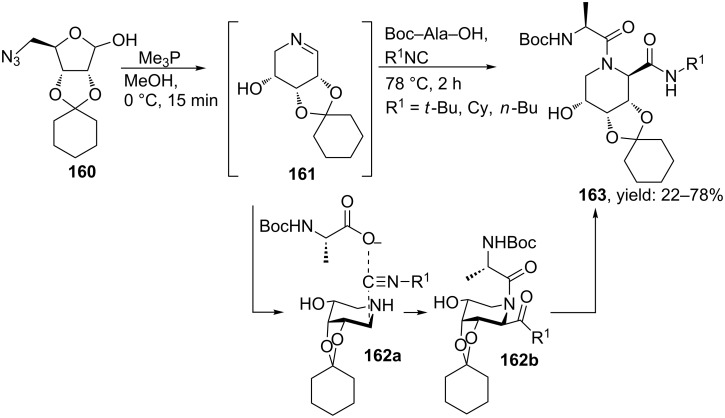
Staudinger–Aza-Wittig/Ugi-approach towards pipecolic acid peptidomimetics.

The group of van Boom and Overkleeft reported pipecolic amides via a Staudinger–Aza-Wittig/Ugi sequence (SAWU 3CR, Scheme 51) [132]. First the Staudinger reaction between an orthogonally protected carbohydrate-derived azido-acetal and trimethylphosphine yielded the necessary cyclic imine, which then was exposed to benzoic acid and different isocyanides. The resulting Ugi-bisamides **163** were obtained in moderate to good yields (22–78%), in which the more sterically demanding isocyanides gave the best results. In addition, the Ugi-products were obtained as single diastereomers with a *trans*-configuration. They argue that the isocyanide and acid substrates react at the least hindered side of the imine.

#### 2,5-Diketopiperazines

**Figure 1:**
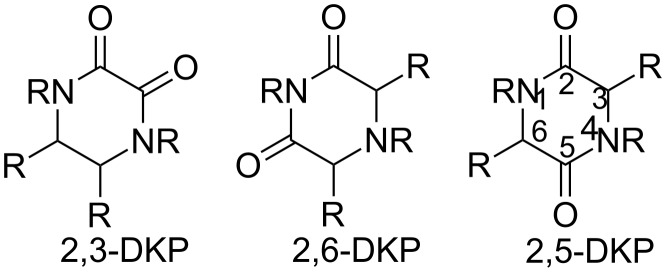
The three structural isomers of diketopiperazines. The 2,5-DKP isomer is most common.

Diketopiperazines (DKPs) are the smallest class of naturally occurring cyclic peptides containing a six-membered core ringsystem. Such DKPs were shown to possess several interesting medicinally relevant properties such as antifungal [133–134], antibacterial [135], and antitumor activity [136–137] but are also used to introduce for example a bitter taste in e.g. beer, cacao and coffee [138–140]. The DKPs occur in three different isomers, in which the position of one oxo-group is different at the piperazine-ring [26]. The 2,5-diketopiperazines are most relevant due to the structural similarity with peptides (Figure 1).

The DKP core shows properties for enhanced interaction with biological targets such as metabolic stability, conformational rigidity and the scaffold can both donate and accept hydrogen bonds. Furthermore, diversity can be introduced at four positions (N1, N4, C3 and C6). An important feature of DKPs is that they are able to induce secondary structures such as β-turns, β-hairpins in β-sheets and α-helices [141]. Therefore, DKPs are often used as peptidomimetic building blocks. A more detailed review of diketopiperazines is published by Borthwick and Piarulli [141–142].

**Scheme 52:**
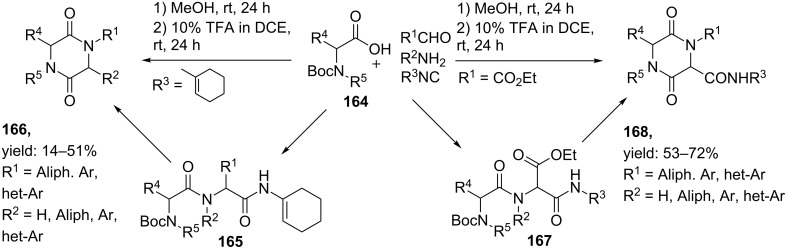
UDC-approach to obtain 2,5-DKPs, either using Armstrong’s isocyanide or via ethylglyoxalate.

Standard multicomponent reactions towards DKPs have the disadvantage that cyclization of the linear dipeptide unit is difficult. Instead of relatively easy ester cyclizations, the Ugi-product contains a C-terminal amide that is more difficult to cyclize. Therefore, Ugi MCRs towards diketopiperazines require subsequent post-condensation modifications such as Ugi-deprotection-cyclizations (UDC), Ugi-activation-cyclizations (UAC) or the combination of both (UDAC). Hulme et al. [143–144] was the first who reported a library of DKPs via a solution phase UDC approach as shown in Scheme 52. An Ugi reaction of Armstrong’s convertible isocyanide, a variety of aldehydes and amines and bifunctional amino acid **164** in methanol afforded the corresponding linear Ugi-products **165** in good yields (72–92%). Subsequent *N*-Boc deprotection and activation of the isocyanide amide in acidic environment allowed cyclization to the diketopiperazines scaffolds **166** (overall 14–51%). As alternative, the authors reported an Ugi reaction with ethylglyoxalate as bifunctional component since in certain cases the convertible isocyanide performed sub-optimal in the cyclization [145]. Via this procedure dipeptide **167** was obtained, that after TFA- treatment in dichloroethane (DCE) resulted in the desired DKPs products **168** in good to excellent yields (53–72%).

**Scheme 53:**
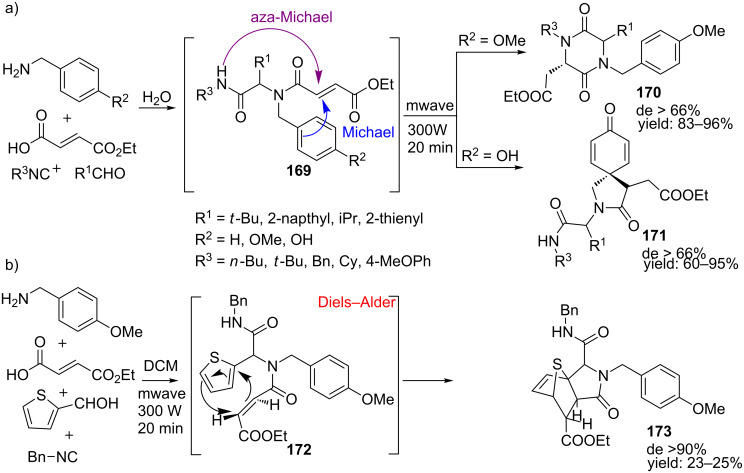
a) Ugi reaction in water gave either 2,5-DKP structures or spiro compounds. b) The Ugi reaction in DCM gave tricyclic lactams.

Solvent effects of the Ugi reaction under microwave irradiation were considered by Santra and Andreana (Scheme 53) [146]. Protic solvents such as water gave rise to either 2.5-diketopiperazines **170** via an aza-Micheal reaction or 2-azaspiro-[4,5]deca-6,9-diene-3,8-diones (**171**) via a 5-*exo*-Michael addition, whereas DCM as solvent induced an intramolecular thiophene Diels–Alder reaction yielding tricyclic lactam **173**.

**Scheme 54:**
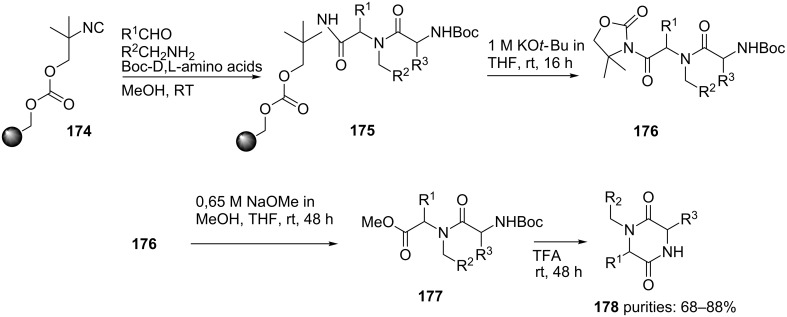
Solid-phase approach towards diketopiperazines.

In an alternative approach an Ugi-protocol employing a resin-bound carbonate-based convertible isocyanide **174** (CCI) was reported [147]. A wide variety of aldehydes, primary amines and carboxylic acids were tolerated resulting in a library of 80 different linear dipeptides. Cleavage from the carbonate resin with KO*t*-Bu afforded compound **176** which was converted to the methyl ester **177** using NaOMe (Scheme 54). Subsequent TFA-treatment resulted in the desired diketopiperazines **178**.

**Scheme 55:**
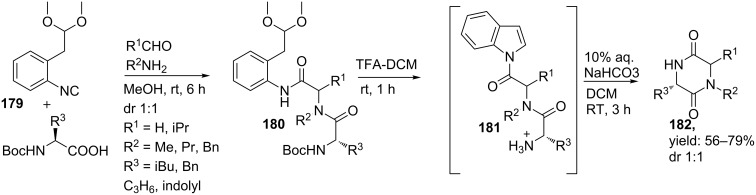
UDAC-approach towards DKPs.

The group of Wessjohann reported a small library of DKPs using the acidic-labile convertible isocyanide **179** [148] in combination with readily available primary amines, aldehydes and *N*-Boc-protected amino acids [140]. It was shown that treatment of the Ugi-adduct **180** with acid both cleaved the *N*-Boc-protecting group and activated the nitrile amide. Subsequent addition of a base induced cyclization and resulted in the DKP-scaffolds (**182,** Scheme 55). In total seven compounds were synthesized based on this UDAC-protocol with yields varying from 56 to 79%.

**Scheme 56:**
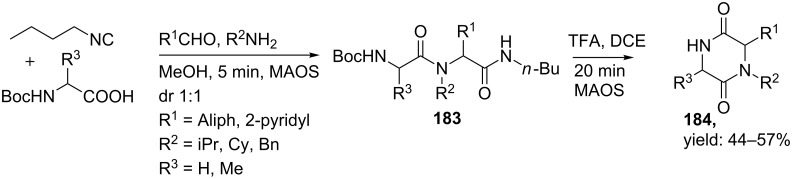
The intermediate amide is activated as leaving group by acid and microwave assisted organic synthesis (MAOS).

Recently, another UDC-based synthesis of DKP scaffolds using the cheaper and commercial available *n*-butylisocyanide as convertible component was reported [149]. The scaffolds were obtained in good yields in a 1:1 diastereomeric ratio. However, microwave heating was required to induce cyclization (Scheme 56).

**Scheme 57:**
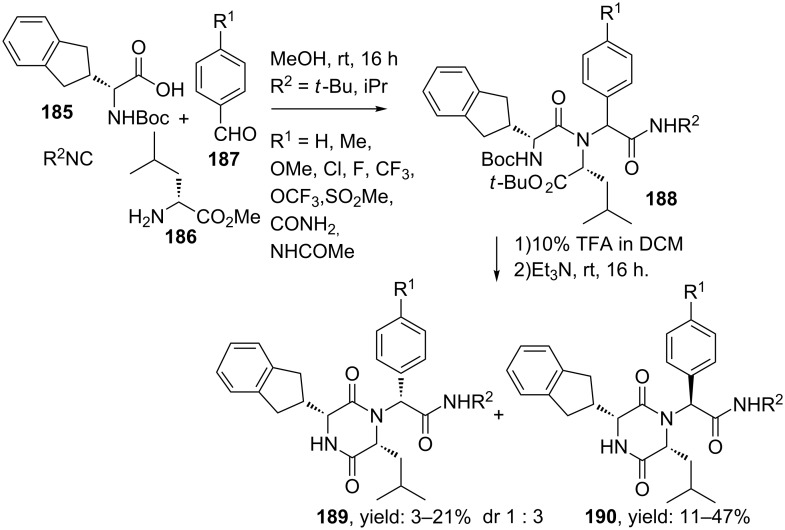
UDC-procedure towards active oxytocin inhibitors.

In addition the UDC-approach was also used for a small library of orally active diketopiperazines active against the oxytocin receptor [150–151]. Rapid access towards these antagonists is highly desirable since inhibition of this receptor delays preterm labour in newborns [152]. The UDC-approach started with an Ugi reaction of aryl aldehydes, isonitriles, D-leucine methyl ester and *N*-Boc-D-indanylglycine (derived from the benzhydrylimine of *N*-Boc-glycine, ee >99%) in methanol and afforded linear dipeptide **188** (Scheme 57). Subsequent treatment with TFA followed by base catalyzed cyclization provided both (3*R*,6*R*,7*R)*- and (3*R*,6*R*,7*S*)-isomers, in favour of the latter (dr 1:3). However, the minor *RRR*-isomers **189** showed to have the highest potency and were obtained in yields up to 21%.

**Scheme 58:**
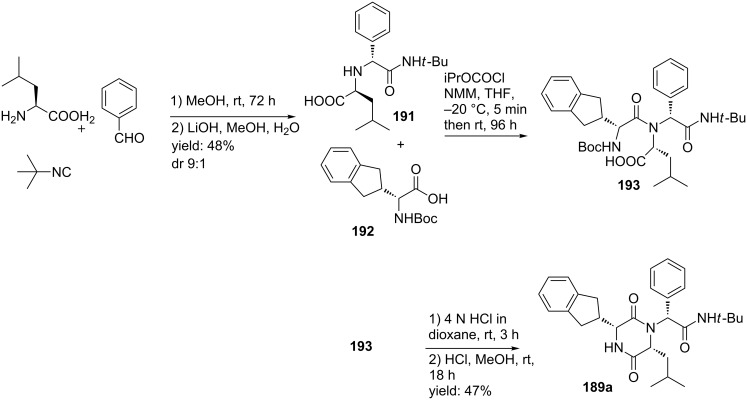
An improved stereoselective MCR-approach towards the oxytocin inhibitor.

In a variation, improved stereoselective reaction for the *RRR*-isomer **189** was observed. After an Ugi 4C-3-CR of benzaldehyde, L-leucine, *t-*butylisonitrile in methanol followed by subsequent hydrolysis of the ester the *RS*-acid **191** was formed in 48% yield (Scheme 58) [153]. The acid was then combined with the in situ-formed anhydride derivative of (*R*)-Boc-indanyl glycine (**192**) and subsequent cyclization resulted in **187** in 47% yield. It is noteworthy that via this particular route, the configuration of the leucyl amide is inverted during the coupling reaction, whereas the chirality of phenyl glycine and the indanyl glycine are retained.

**Scheme 59:**
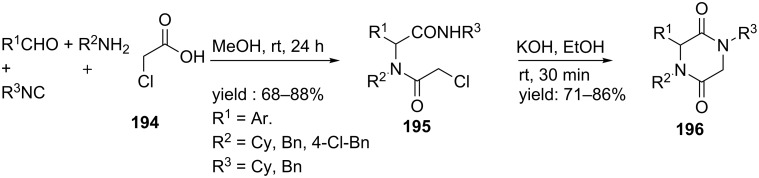
The less common Ugi reaction towards DKPs, involving a S_n_2-substitution.

A less common approach was developed by Marcaccini and co-workers [154]. They obtained 2,5-DKPs in high yields by reacting 2-chloroacetic acid **194** with different aromatic amines, isocyanides and aldehydes in methanol followed by cyclization in ethanolic KOH under ultrasonic conditions (Scheme 59).

#### Bicyclic diketopiperazines

**Figure 2:**
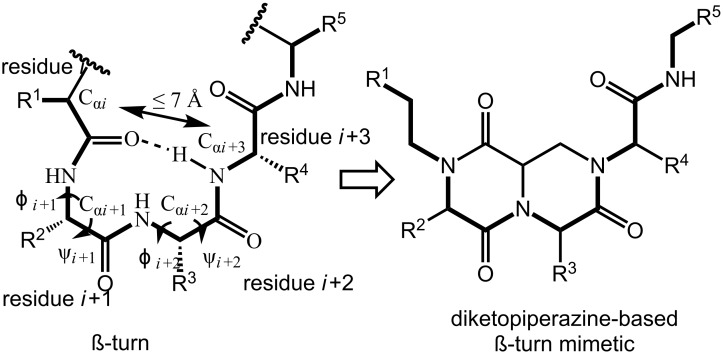
Spatial similarities between a natural β-turn conformation and a DKP based β-turn mimetic [158].

The development of bicyclic diketopiperazines has received special interest since these scaffolds force the molecule into a similar conformation as the type I β-turn in native peptides [142,155]. Therefore, β-turn mimetics based on this bicyclic core can reveal important information about the biologically active conformation of the native peptide [69,155]. β-Turns are characterized as any tetrapeptide sequence which is stabilized by an intramolecular H-bond between residue *ί* and *ί*+3 forming a pseudo-ten-membered ring [10,156]. The distance between the α-carbons of these two residues is ≤ 7 Å (Figure 2) [157].

**Scheme 60:**
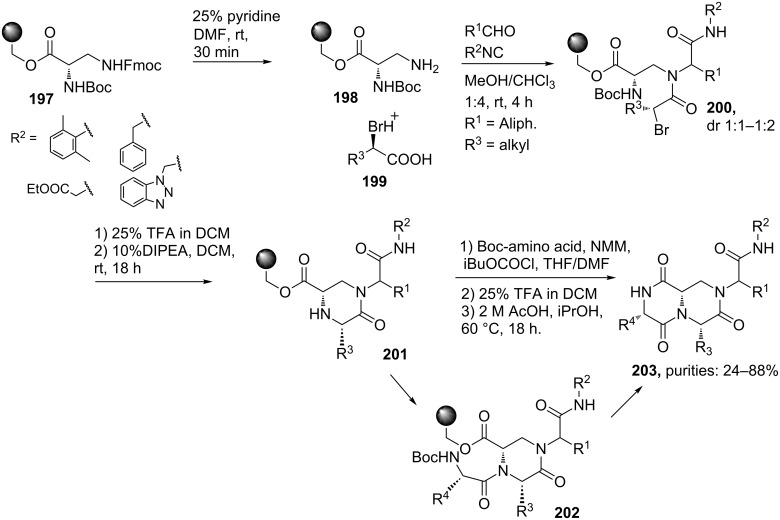
Ugi-based syntheses of bicyclic DKPs. The amine component is derived from a coupling between (*R*)-*N*-Boc-α-*N*-Fmoc-*L*-diaminopropionic acid and Merrifield’s hydroxymethyl resin under Mitsunobu conditions, followed by a standard Fmoc deprotection.

There are two different types of β-turn mimetics possible, external and internal mimics [159]. The former includes turn-inducing-scaffolds that in most cases replace the *ί*+1 and *ί*+2 residues and have their rigidifying moiety lying outside the hydrogen bonded ring. Examples are lactams and dihydropyridimidinones .In contrast, internal mimics have their rigidifying part lying in the pseudo-ten-membered ring. Examples are bicyclic scaffolds such as diketopiperazines. A multicomponent approach to these latter scaffolds is described by Golebiowski et al. (Scheme 60) [156,160]. Herein, the Ugi reaction involving resin-bound amine **198** and an excess of *R*-(+)-2-bromoalkyl acid **199**, isocyanide and aldehyde (5 equiv) afforded the linear dipeptide **200** that after acidic Boc-removal and base-catalyzed S_n_2-cyclization was converted to the monocyclic ketopiperazine **201**. The authors coupled this modified Ugi-adduct to different *N*-Boc-amino acids, in which TFA treatment and subsequent cyclization in acetic acid furnished the bicyclic diketopiperazines **203**. During the Ugi reaction, inversion of configuration was observed at the R^3^-position (from bromine displacement by a S_n_2-mechanism), whereas the stereochemistry at the central bridging carbon originates from the chirality of diaminopropionic acid, derived from either L- or D-asparagine. The scope of the Ugi reaction includes several aliphatic and aromatic aldehydes, in which the former gave higher conversions. However, only a limited set of isocyanides were tolerated in this approach.

#### Other bicyclic derivatives

**Scheme 61:**
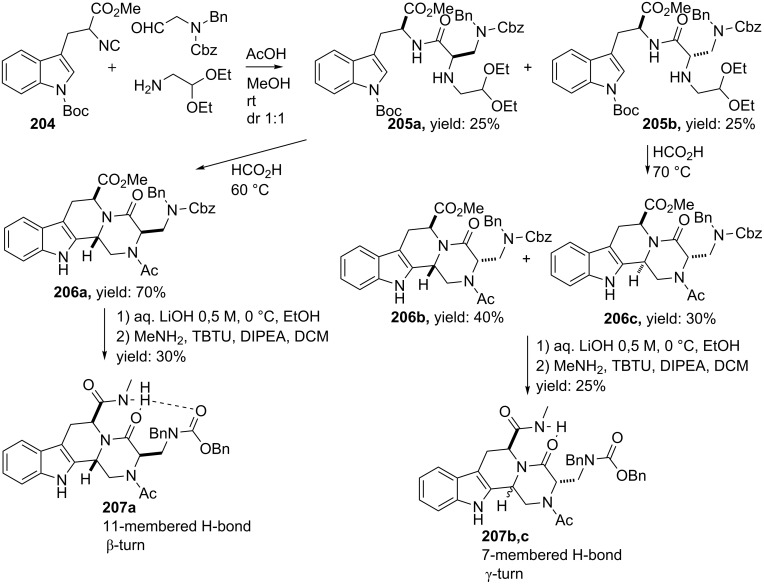
Ugi-based synthesis of β-turn and γ-turn mimetics.

As an alternative to (bicyclic) DKPs, the group of Silvani reported a tetracyclic tetrahydro-β-carboline (THBC)-based turn-mimic via an Ugi/Pictet–Spengler combination [16]. The Ugi reaction provided two diastereomers (**205a**,**b**, dr 1:1), both in 25% yield by reacting *N*-diprotected-2-aminoacetaldehyde (used for the first time in an Ugi-like reaction), *N*-protected tryptophan derived isocyanide **204**, aminoacetaldehyde diethyl acetal and acetic acid (Scheme 61). The subsequent Pictet–Spengler reaction provided three steroisomers **206a**,**c**. To investigate the turn-properties, the authors converted the products to the corresponding carboxamide *N*-acetyl analogues via a hydrolysis and subsequent condensation with MeNH_2_. Both NMR and modelling studies confirmed the formation of a β-turn like conformation for the *cis*-isomer **207a** and γ-turns for the *trans*-isomers **207b**,**c**.

#### 3,4-Dihydropyridin-2-ones

**Figure 3:**
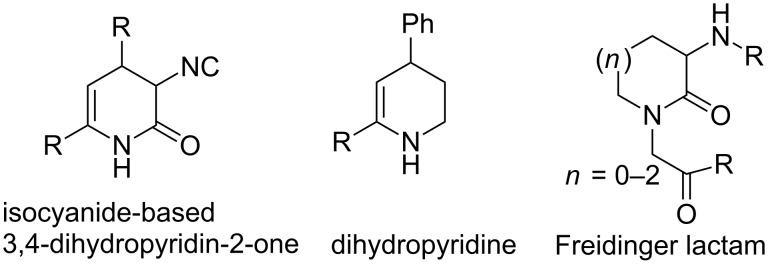
Isocyanide substituted 3,4-dihydropyridin-2-ones, dihydropyridines and the Freidinger lactams. Bio-active calcium channel modulators, the dihydropyridines should contain an axial phenyl substituent at the C4-center adopting a boat conformation.

Another interesting class of 6-membered heterocyclic rings that can be used in peptidomimetics is the 3,4-dihydropyridin-2-one. Conformationally, dihydropyridin-2-ones can be compared to dihydropyridines (DHP), which in turn have shown potential as calcium channel modulators [161–162]. Furthermore, these scaffolds have structural similarities with Freidinger lactams (Figure 3) [161–162].

**Scheme 62:**
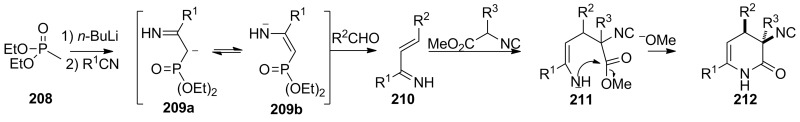
The mechanism of the 4-CR towards 3,4-dihydropyridine-2-ones **212**.

In 2007, our group reported the synthesis of 3,4-dihydropyridin-2-ones via a double MCR approach [163–164]. The first MCR provided the 3,4-dihydropyridin-2-one core by reacting phosphonate **208** with various nitriles, aldehydes and α-aryl isocyanoacetates. This particular 4-CR involves a Horner–Wadsworth–Emmons (HWE) reaction, in which first the phosphonate is deprotonated [125]. Subsequent addition to the nitrile-component resulted in the ketimine intermediate **209a**,**b** which is more nucleophilic at carbon than at nitrogen and reacts with the aldehyde, generating an in situ 1-azadiene intermediate **210**. A subsequent Michael attack by the isocyanide α-carbon atom, followed by a lactonization resulted in the core structure containing an isocyanide moiety (**212,** Scheme 62).

**Scheme 63:**
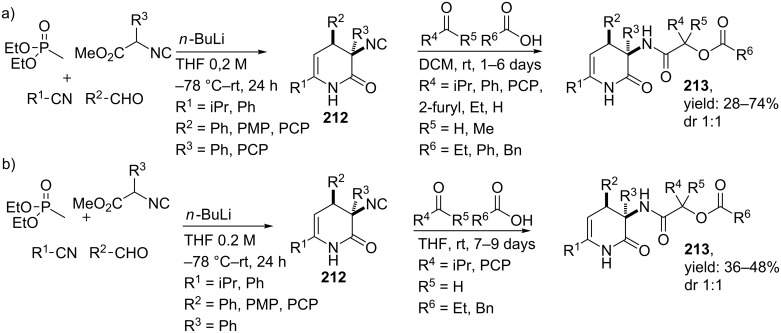
a) Multiple MCR-approach to provide DHP-peptidomimetic in two-steps. b) A one-pot 6-CR providing the same compounds.

Variation of all substrates except the phosphonate proved the possible formation of the isonitrile-functionalized 3,4-dihydropyridin-2-ones in good yields, in which aromatic isocyanoacetates exclusively gave the *cis*-diastereomer. In addition, aliphatic isocyanoacetates only show a preference for the *cis*-diastereomer if the cyclization step was performed at higher temperatures [12]. We argued that epimerization (of the C4-center) to the more thermodynamically stable isomer was the reason for this. More interestingly, the isocyanide moiety did not react and was left intact during the initial 1-azadiene-based multicomponent reaction. This opened the way for an additional Passerini 3-CR (Scheme 63), in which a wide variety of aldehydes/ketones and acids successfully reacted with the isocyanide to obtain depsipeptides **213** in overall yields of 28–74% (dr 1:1). In a variation, we combined both MCRs to a one-pot 6-CR, and obtained the depsipeptides in comparable yields as the two-step procedure.

**Scheme 64:**
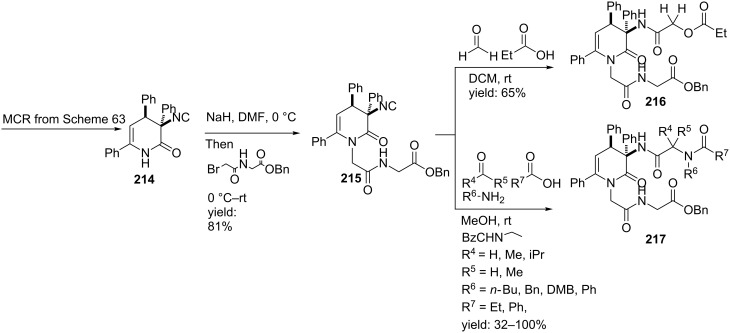
The MCR–alkylation–MCR procedure to obtain either tetrapeptoids or depsipeptides.

Moreover, the structural similarities of C3-substituted 3,4-dihydropyridin-2-ones with Freidinger lactams inspired our group to investigate possible turn properties of this restricted core element [10]. Since modelling studies confirmed that these scaffolds can adopt type IV β-turn structures, we developed constrained tetra/penta (depsi) peptides via a quick MCR–alkylation–MCR approach. It is noteworthy that both the Passerini and the Ugi reaction could be applied as second MCR, providing the cyclic constrained peptide-like structures in good yields (Scheme 64). As an extension, we also incorporated *N*-protected amino acids as acid input in order to provide penta(depsi)peptides **216** and **217**. Unfortunately, based on spectroscopic analyzes (X-ray crystallography and ^1^H NMR) none of these penta or tetra mimics adopted a true β-turn conformation. Nevertheless, these scaffolds consist of rigidifying properties and can be used as conformationally constrained building blocks in the design of peptidomimetics.

#### Triazines

**Scheme 65:**
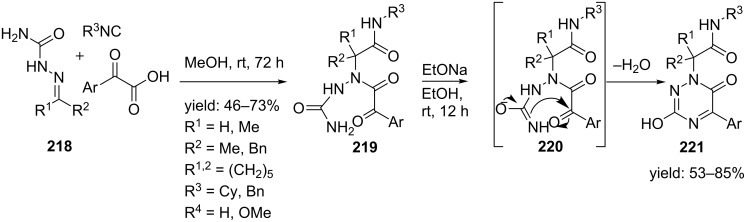
U-3CR/cyclization employing semicarbazone as imine component gave triazine based peptidomimetics.

Aza- and urea-based peptidomimetics have shown to be useful peptide isosteres in several therapeutic applications [166–168]. In addition, their cyclic constraints such as 1,2,4-triazines can induce peptide-turns and according to literature 1,2,4-triazines are active as selective herbicides [169], HIV-protease inhibitors [170] and anti-cancer agents [171–173]. However, multicomponent reactions towards them are scarce. The group of Torroba and Marcacinni reported an interesting Ugi 3-CR/cyclization approach towards pseudopeptidic 6-oxo-[1,2,4]-triazines (Scheme 65) [174]. The linear Ugi-adducts **219** were obtained from a reaction between phenylglyoxalic acid, several isocyanides and semicarbazone **218** as imine component, in which the incorporation of the latter was not reported before. Addition of sodium ethoxide in ethanol promoted cyclization and afforded the triazines **221** in good overall yields.

**Scheme 66:**
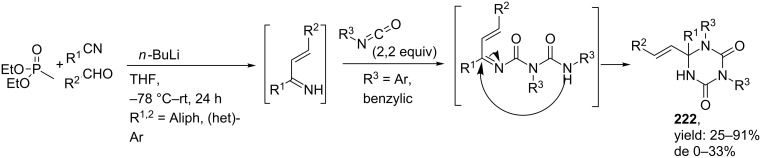
4CR towards triazinane-diones.

**Scheme 67:**
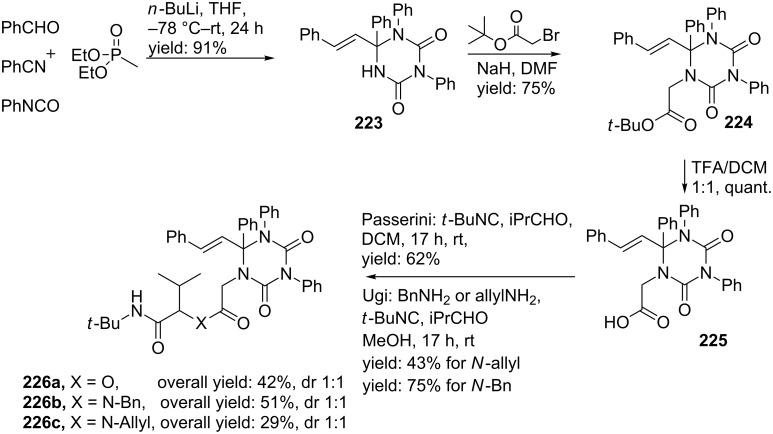
The MCR–alkylation–IMCR-sequence described by our group towards triazinane dione-based peptidomimetics.

Our group published the synthesis of triazinane diones as novel cyclic urea derivatives via a 4-CR-alkylation-IMCR sequence [165,175–177]. The 4-CR involves the HWE reaction described above between a phosphonate, nitrile and aldehyde, in which the in situ-formed 1-azadiene is trapped by an isocyanate (instead of an isocyanoacetate) to afford the triazinane dione core (**222**, Scheme 66). For the scope of this reaction a wide range of aliphatic and (hetero)aromatic nitriles and aldehydes and several benzylic and aromatic isocyanates were tolerated, whereas for the phosphonate input only diethyl methyl- or ethylphosphonate were compatible. From the results it became clear, that addition of 2,2 equivalents of the isocyanate was favourable for the reaction and increased the yield of **223** up to 91%. A subsequent *N*-alkylation with *tert*-butyl 2-bromoacetate followed by deprotection of the *tert*-butyl group furnished the carboxylic acid **225** which could further react in an additional Ugi or Passerini reaction (Scheme 67). The Passerini reaction was performed with isobutyraldehyde, acid **225** and *tert*-butyl isocyanide to provide the depsipeptide-like product **226a** in 62% yield, whereas the Ugi reaction was employed with the same substrates and benzyl– or allyl amine as fourth component to provide two peptidoyl triazinane diones **226b,c** in 43% and 75% yield for the last step, respectively [176].

#### Other 6-membered ring constraints

**Scheme 68:**
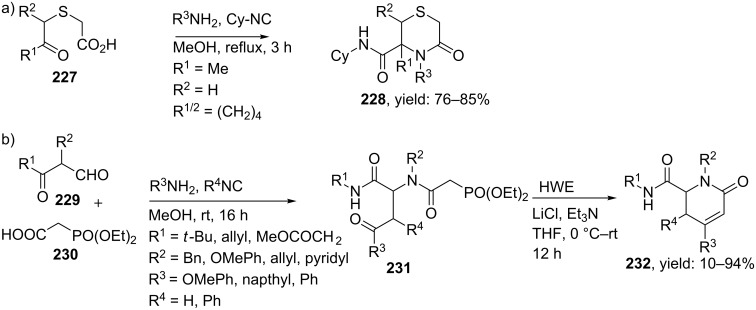
Ugi-4CR approaches followed by a cyclization to thiomorpholin-ones (a) and pyrrolidines (b).

In addition to a range of MCR-based protocols available for the above discussed six-membered ring constraints, a few other types of (hetero) cyclic peptidomimetics containing a six membered ring have been reported. Among them, the thiomorpholin-3-one heterocycle is used in several therapeutic applications [178–179] and an Ugi-based MCR was reported by the group of Marcaccini (Scheme 68) [180]. In this work, monocyclic and bicyclic 5-oxothiomorpholine-3-carboxamides **228** were obtained in 76–85% yields (dr 1:1) by reacting bifunctional oxoacids **227**, benzylamines and cyclohexyl isocyanides in methanol. Pyrrolidone-constrained peptidomimetics can be obtained via an Ugi/HWE sequence as describe by Dömling et al. [181]. They obtained the linear Ugi-products **231** by reacting α-keto aldehydes **229**, phosphono acetic acid **230** and a variety of isocyanides and primary amines in methanol. The following HWE reaction was performed under basic conditions and furnished the 6-oxo-1,2,3,6-tetrahydro-pyridine-2-carboxylic acid amides **232** in modest to excellent yields (10–94%).

#### Seven membered ring constraints

**Benzodiazepines**

**Scheme 69:**
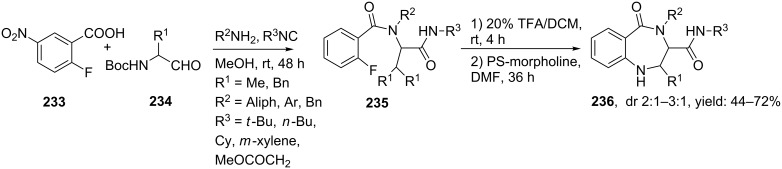
UDC-approach for benzodiazepinones.

Benzodiazepines (BDPs) represent an important class of small seven membered ring peptidomimetics. These BDPs demonstrate numerous therapeutic applications ranging from protease inhibitors against HIV [182] and malaria [183–184] to drugs with anticancer [185–187] or psychoactive properties [188]. In addition, the diazepinone ring also possesses turn and α-helix inducing properties 8 [189–191]. Multicomponent approaches towards BDPs usually comprise an Ugi reaction along with several cyclization strategies. Hulme and co-workers reported an UDC strategy involving a S_n_Ar cyclization (Scheme 69) [192]. The Ugi products herein were obtained in good yields by reacting 2-fluoro-5-nitrobenzoic acid **233** with *N*-Boc-α-amino aldehydes **234** and several isocyanides and amines. Subsequent TFA treatment and cyclization induced by a proton scavenger revealed a library of 80 BDPs (**236**, 44–72%, dr 2:1–3:1).

**Scheme 70:**
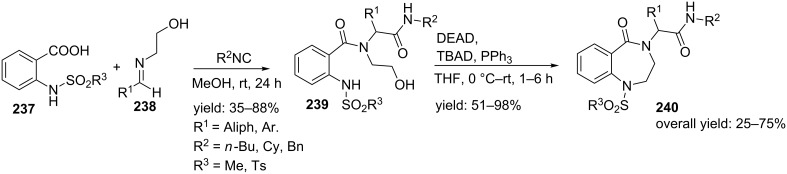
Ugi/Mitsunobu sequence to BDPs.

Banfi et al. published an Ugi/Mitsunobu combination towards sulfonamide-based BDPs, **240** which makes use of imines **238**, isocyanides and acid **237** [193]. Herein, the imines were obtained from aldehydes and ethanolamine. The subsequent cyclization using standard Mitsunobu conditions furnished the BDPs in good overall yields (Scheme 70).

**Scheme 71:**
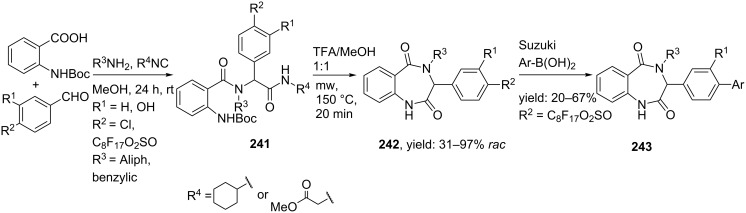
A UDAC-approach to BDPs with convertible isocyanides. The corresponding amide is cleaved by microwave heating, thereby providing the 7-membered ring.

A microwave-mediated UDAC-procedure employing convertible isocyanides was also reported (Scheme 71) [194]. The usually non-convertible cyclohexylamino- and methylacetylamino isocyanides proved in this case ideally suited as convertible substrates. The Ugi-products were obtained by combining these isocyanides with a variety of aromatic aldehydes, bifunctional acids and both aliphatic and benzylic amines. Subsequent *N*-Boc-deprotection and microwave-assisted cyclization furnished a small library of BDPs (**242**, yields 31–97%). In addition, it was also shown that fluoro-benzaldehydes allow further scaffold derivatization via a subsequent Suzuki coupling.

**Scheme 72:**
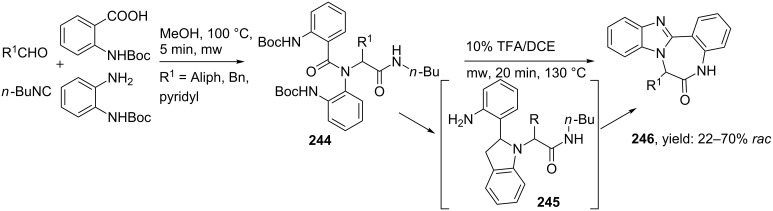
microwave assisted post condensation Ugi reaction.

In a variation, Hulme et al. developed a similar approach utilizing two internal nucleophiles towards tetracyclic BDPs (Scheme 72) [195]. Deprotection of the Ugi-products activated the nitrile functionality and unmasked both amino-groups, in which microwave irradiation allowed a sequential double cyclization to the tetracyclic benzimidazole-benzodiazepines **246**. During these cyclizations the authors observed that the order of cyclization was in favour of the benzimidazole, nonetheless after 20 minutes of irradiation all the intermediates were converted to the tetracyclic scaffolds in modest to high yields (22–70%).

**Scheme 73:**
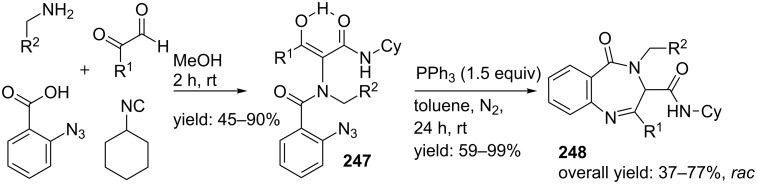
Benzodiazepinones synthesized via the post-condensation Ugi/ Staudinger–Aza-Wittig cyclization.

β-Turn mimetics of type **248** were developed by the groups of Marcaccini and Torroba [196] via an Ugi/Staudinger–Aza-Wittig sequence (Scheme 73). The Ugi reaction of arylglyoxals, *para*-substituted benzylamines, cyclohexyl isocyanide, and 2-azidobenzoic acid provided the linear dipeptides **247**. Subsequent addition of triphenylphopshine induced a Staudinger–aza-Wittig cyclization and furnished the BDPs **248** in 37–77% overall yields. From spectroscopic studies it became clear that these conformationally restricted peptidomimetics adopt type I, I’, II and II’ β-turn conformations.

**Scheme 74:**
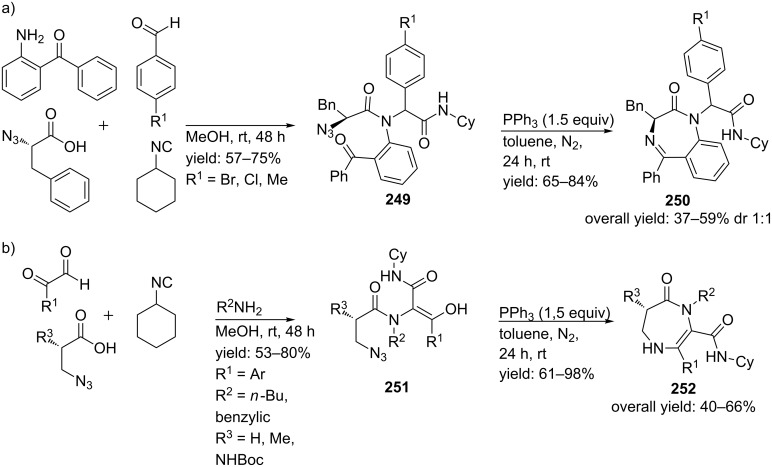
Two Ugi/cyclization approaches utilizing chiral carboxylic acids. Reaction (a) provided the products in a diastereomeric mixture of 1:1, whereas reaction (b) yielded the products as single enantiomers.

In 2010, the same groups performed the Ugi reaction with (*S*)-3-phenyl-2-azidopropionic acid (instead of 2-azidobenzoic acid) in order to control stereochemistry at the position of the aryl glycine moiety (Scheme 74) [197]. However, no stereoinduction was observed at this stereocenter and the BDPs **250** were obtained as mixtures of diastereomers (dr 1:1, 37–59%). In the same report, they described an enantioselective Ugi/cyclization reaction in which monocyclic diazepinones **252** were obtained as single *S*-enantiomers (40–66%). In this approach the Ugi reaction was performed with optically pure (*S*)-3-azidopropionic acids and 2-aminobenzophenone (Scheme 74).

**Other seven membered ring derivatives**

**Scheme 75:**
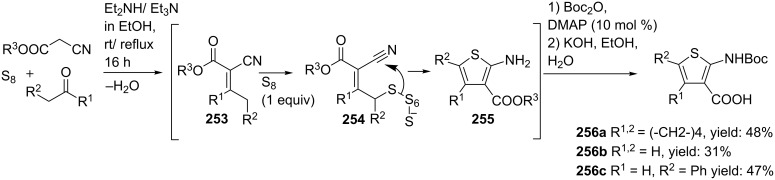
The mechanism of the Gewald-3CR includes three base-catalysed steps involving first a Knoevnagel–Cope condensation between α-methylene carbonyls and α-activated acetonitriles, second an addition of sulfur to the α-β-unsaturated intermediate and third a cyclization towards the 2-aminothiophene.

**Scheme 76:**
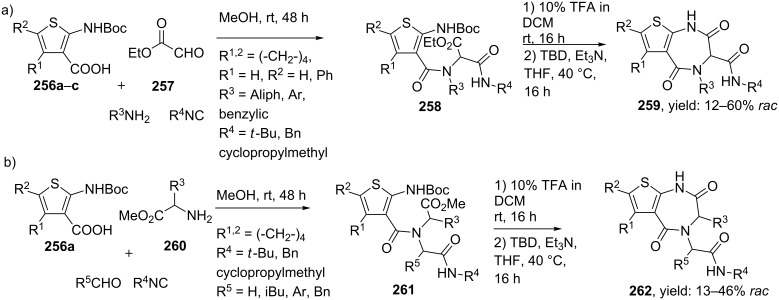
Two structural 1,4-thienodiazepine-2,5-dione isomers by U-4CR/cyclization.

Dömling and co-workers reported a convenient route towards 1,4-thienodiazepine-2,5-diones [198]. Thiophenes can be synthesized via the Gewald 3-CR, providing 2-aminothiophenes, which in turn have shown to be suitable derivatives of anthranilic acids (Scheme 75) [199–200]. This inspired the researchers to combine the Gewald 3-CR with a sequential Ugi-deprotection–cyclization in order to obtain 1,4-thienodiazepine-2,5-diones. The Ugi reaction of **256**, **257** and a variety of amines and isocyanides gave access to the linear dipeptides **258**. Subsequent TFA-deprotection and cyclization catalyzed by the strong guanidine base triazabicyclodecene (TBD) afforded the 1,4-thienodiazepine-2,5-diones **259** in moderate to excellent overall yields (12–60%, Scheme 76). Additional modelling studies showed that these mimics consist of α-helix-inducing properties and that they can be used as potent tumor suppressors. In a variation, the authors shifted the (*exo-*) peptide chain from carbon to the neighbouring nitrogen by performing the Ugi reaction with different amino esters as amine source (Scheme 76) [201].

**Scheme 77:**
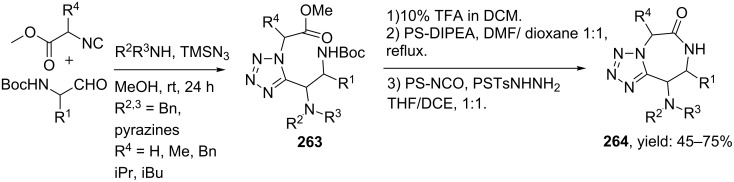
Tetrazole-based diazepinones by UDC-procedure.

Tetrazole-based diazepinones were obtained via a TMSN_3_-modified UDC protocol reported by Hulme and co-workers (Scheme 77) [202]. A variety of secondary amines, *N*-Boc-amino-aldehydes, and substituted methylisocyanoacetates were tolerated and provided the tetrazoles **263** in good yields. TFA treatment and the addition of a proton scavenger allowed cyclization and furnished the tetrazole-diazepine-ones **264** in 45–75% yield.

**Scheme 78:**
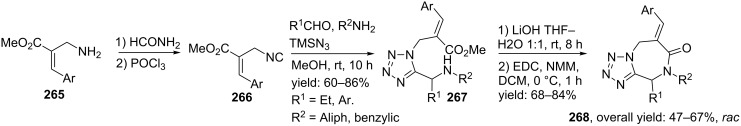
Tetrazole-based BDPs via a sequential Ugi/hydrolysis/coupling.

In a variation, Nayak and Batra reported an Ugi/hydrolysis/coupling sequence starting from allyl isonitrile **266** that was synthesized from its corresponding primary allyl amine **265**, which in turn was derived from Baylis–Hillsman acrylates [203]. The tetrazole-based Ugi adducts **267** were obtained in high to excellent yields (60–86%), that via subsequent ester-hydrolysis and coupling with EDC and NMM resulted in the tetrazole-based BDPs **268** in overall yields of 47–67% (Scheme 78).

**Scheme 79:**
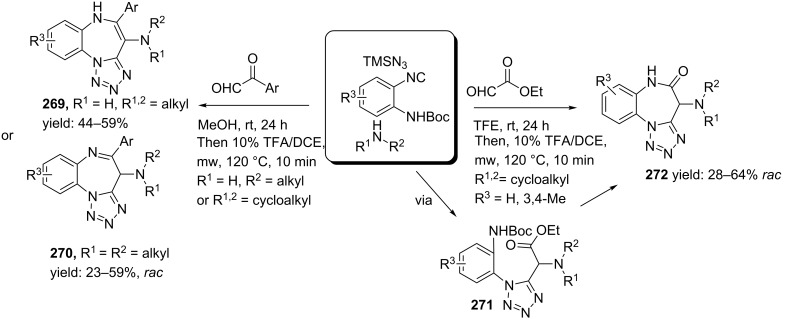
MCR synthesis of three different tricyclic BPDs.

Tricyclic tetrazole-fused BDP derivatives were reported as well (Scheme 79) [204]. In this case an Ugi-Azide reaction using amines with ethylglyoxalate, TMSN_3_ and bifunctional isocyanide in trifluoroethanol were employed to obtain **271**. An additional Boc-cleavage and cyclization under microwave conditions afforded the benzotetrazolediazepinones **272**. As an extension, the authors also performed the Ugi reaction with arylglyoxaldehydes together with either primary or secondary amines, in which the primary amines exclusively led to benzotetrazolodiazepines **269**, whereas incorporation of secondary amines afforded either benzotetrazolodiazepines **269** or **270**. However, these latter analogues are prone to hydrolysis and oxidation at room temperature.

**Scheme 80:**
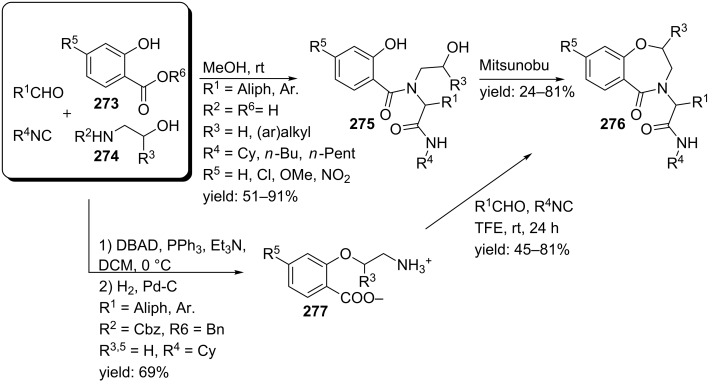
Two similar approaches both involving an Ugi reaction and a Mitsunobu cyclization.

Another important structural motif that can constrain peptides is the 1,4-oxazepine [205–208]. Only a few multicomponent approaches have been described towards 1,4-oxazepine analogues. For example, dihydro-1,4-benzoxazepines and dihydro-1,4-benzoxazepin-5-ones have been reported by Banfi et al. [209]. The dihydro-1,4-benzoxazepin-5-ones **276** were synthesized by either a sequential Ugi–Mitsunobu cyclization or employing a reversed version of the sequence (Mitsunobu–Ugi). Both procedures gave similar results, however, the latter one required an additional deprotection step (Scheme 80). In addition, the Mitsunobu reactions were performed with PPh_3_ and DBAD. The scope of the Ugi reaction tolerated a wide variety of isocyanides and aldehydes, affording the bicyclic scaffolds in good yields. Furthermore, additional modelling studies revealed that these mimics could induce α-helix-conformations, when the R^1^, R^3^, R^5^ substituents contains (aryl)alkylchains [190].

**Scheme 81:**
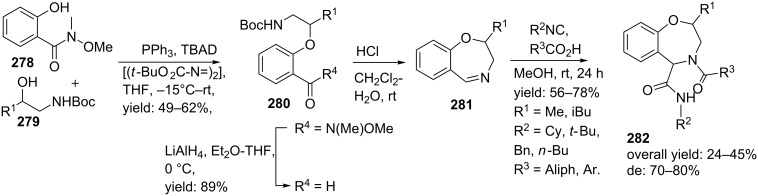
Mitsunobu–Ugi-approach towards dihydro-1,4-benzoxazepines.

In contrast, dihydro-1,4-benzoxazepines **282** (Scheme 81) could be obtained in four-steps by first performing the Mitsunobu reaction with racemic alcohols and the Weinreb hydroxamate **278**. Subsequent reduction and deprotection resulted in the cyclic imines **281** [210]. Then an additional Joullié–Ugi reaction provided the final bicyclic mimics **282** in good to excellent yields, (24–45%) with a preference for the *cis*-isomer. Steric arguments account for the observed selectivities.

**Scheme 82:**
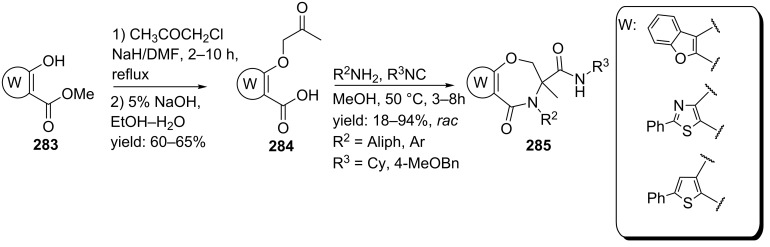
Ugi reaction towards hetero-aryl fused 5-oxo-1,4-oxazepines.

Heteroaryl-fused 5-oxo-1,4-oxazepines have been reported by Ivachtchenko and co-workers (Scheme 82) [211]. The key substrate in their approach employs the bifunctional keto-acid **284**, derived from hydroxy-substituted heteroaryl carboxylates **283**, which in turn were commercially or synthetically available. In total a medium-sized library of 23 heteroaryl-derivatives **285** was developed using three different hetero-aryl keto-acids and a wide variety of amines (18–94%).

#### Nine-membered ring constraints

**Scheme 83:**
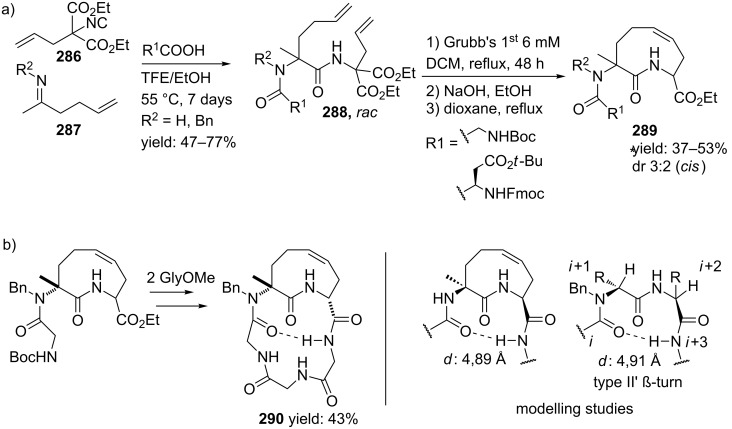
a) Ugi/RCM-approach towards nine-membered peptidomimetics b) Sequential peptide-coupling, deprotection, peptide coupling revealed the GGG-pentacyclic structure that adopts a β-turn conformation.

Multicomponent reactions towards medium-sized cyclic peptidomimics (9–12 membered) usually involve two unsaturated components that can be cyclized via a post ring closing metathesis (RCM). Following this two-step sequence, Banfi and co-workers [212] reported a small library of nine-membered lactams with potential turn-properties (Scheme 83). The isocyanoacetate **286** and the (preformed) imine **287** provided the olefin moieties in the racemic Ugi-products. Subsequent treatment of these Ugi-products **288** with Grubb’s catalyst (first generation) provided the cyclic constructs exclusively in the *Z*-conformation, along with several acyclic dimers as byproducts. Final saponification and decarboxylation, furnished the nine-membered lactams **289** in good yields (37–53%, dr 3:2 for the *cis*-isomer). In order to investigate the turn-properties, the authors coupled the lactam (R^1^ = (Boc)NHCH_2_) analogues with two glycine methylesters that after deprotection and a final peptide-coupling with BOP afforded the pentacyclic structure **290** as shown in Scheme 83. It is noteworthy that only the *cis*-isomer was able to cyclize and was obtained in a reasonable overall yield (43%). In addition, modelling and spectroscopic studies of the structures revealed that these bicyclic scaffolds can adopt a type II’ β-turn motif, in which a hydrogen bond between residue *i* and *i*+3 is formed [11].

**Scheme 84:**
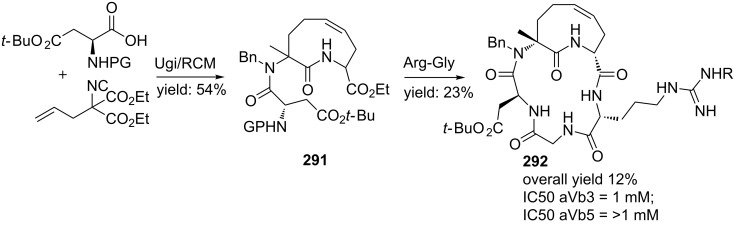
Ugi-based synthesis towards cyclic RGD-pentapeptides.

A second application extended the scope to cyclic RGD pentapeptides (Scheme 84). Peptides containing the RGD-sequence (arginine-glycine-apartic acid) are of great pharmaceutical interest since this tripeptide sequence can be recognized by a special category of receptors, the so-called integrins. Integrins consist of one α- and one β-subunit that play key roles in several biological functions of mammals, for example in cell–cell interactions. Some of them are also involved in the regulation processes of diseases, in which the α_V_β_3_ and α_V_β_5_ integrins are believed to be involved in tumor induced angiogenesis [213–216]. Therefore, inhibition of these integrins by small peptides that contain a RGD-sequence is of high interest [217]. For the development of the cyclic RGD-pentapeptides the authors employed the Ugi/RCM/decarboxylation/coupling sequence, in which the RGD mimics **291** were obtained in overall yields of 12%. In this procedure, the final peptide-coupling was performed with protected Arg-Gly-dipeptide and HATU as couplings reagent [218]. To validate the potency of these mimics, the authors screened their mimics against α_V_β_3_ and α_V_β_5_ integrins and it was shown that the *cis*-isomer was a potent inhibitor of the α_V_β_3_ (IC_50_ = 1 μM) and very weak against α_V_β_5_ (>1 mM).

### Macrocycles

An alternative approach to reduce the flexibility in a peptide backbone and thus limit the number of possible active conformations employs macrocyclization (>12 membered rings) strategies. Such macrocyclic peptides have the additional advantage that no terminal groups are present, which makes them more stable (compared to linear analogues) towards degradation i.e. terminal groups are usually required for efficient protease-binding. In addition, the increased conformational order also reduces the folding-energy required for binding as compared to the flexible linear peptide or mimics [26,219–222]. In order to obtain such peptidic macrocycles via multicomponent reactions, several research groups have utilized post-MCR cyclization reactions such as RCMs, macrolactonizations or nucleophilic aromatic substitutions (S_n_Ar). Alternatively, macrocycle synthesis via cyclizing multiple MCRs have been reported. In this part of the review first the post-MCR-condensations via RCM, lactonization and S_n_Ar will be considered, after that the multiple MCR-approaches will be discussed.

#### Macrocycles via MCR-RCM-approaches

**Scheme 85:**
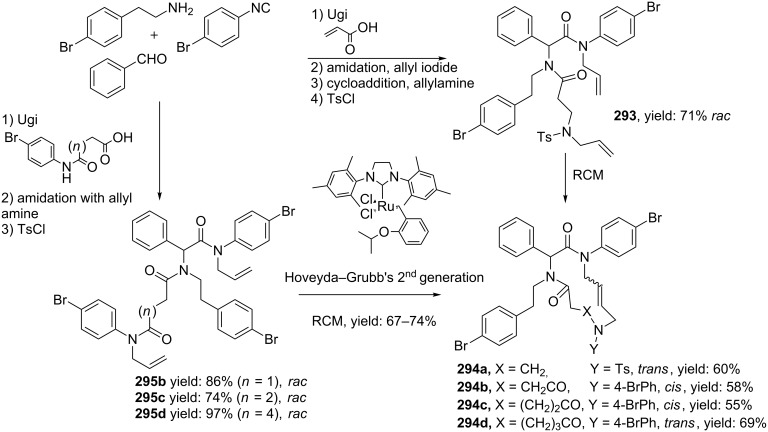
Ugi/MCR-approach towards 12–15 membered macrocycles.

In the past decade three research groups have developed peptide-like macrocycles via a MCR-RCM-sequence. Oikawa et al. reported a small library consisting of 12–15 membered cyclic peptidomimetics [223]. These mimics were synthesized in good yields via a three-step procedure involving an Ugi 4-CR, incorporation of the alkene functionalities and a subsequent RCM assisted by the second generation Hoveyda-Grubb’s catalyst (Scheme 85). Depending on the acid component, the alkene moieties could be introduced by performing either a double amidation with allyl iodide yielding **295** or via a sequential cycloaddition/amidation with allyl amine and allyliodide, respectively, to afford **293**. The follow-up RCM yielded the macrocycles as *trans*-isomer for the 12- and 15-membered cycles and as *cis*-isomer for the 13- and 14-membered cycles **294a–d**.

**Scheme 86:**
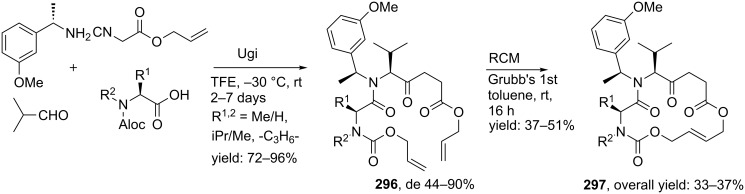
Stereoselective Ugi/RCM approach towards 16-membered macrocycles.

Kazmaier et al, also active in this area, reported a stereoselective Ugi/RCM-approach by utilizing allyl isocyanoacetate and (four) different Alloc-amino acids as bifunctional substrates (Scheme 86) [224]. Whith (*S*)-amino acids and (*S*)-2,2-(*m*-methoxyphenyl)ethylamine as chiral components, the Ugi-products **296** were obtained mainly as the (*S,S,S*)-diastereomers (de 44–90%). Subsequent RCM with Grubb’s 1^st^ generation catalyst afforded the 16-membered macrocycles **297** in good overall yields, favouring the *E*-isomer.

**Scheme 87:**
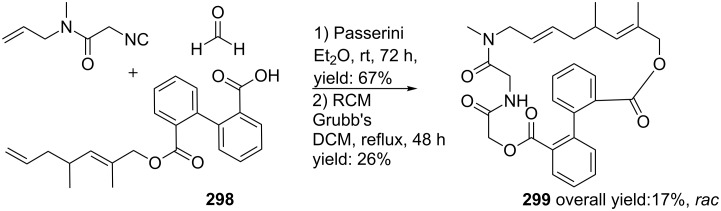
Passerini/RCM-sequence to 22-membered macrocycles.

As an alternative, Dömling et al. applied a Passerini/RCM-approach for the construction of macrocycle **299** (Scheme 87) [225]. Both the carboxylic acid and the isocyanide substrate bear the alkene moiety and were derived from commercially available precursors. The addition of paraformaldehyde provided the linear Passerini-adduct in 67% yield, in which the post-RCM with Grubb’s catalyst afforded the 22-membered macrocycle **299** in 17% overall yield.

#### Macrocycles via MCR-macrolactonization protocols

**Scheme 88:**
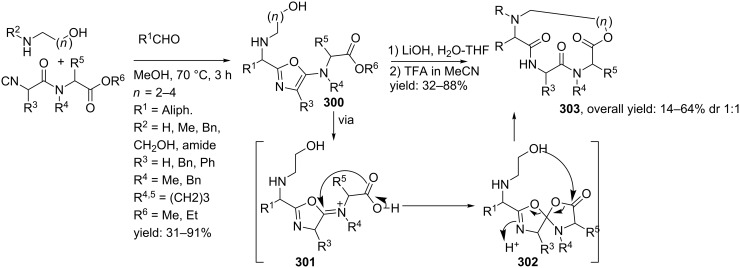
UDAC-approach towards 12–18-membered depsipeptides.

Macrolactonization was employed by Zhu and co-workers after the MCR described above in Scheme 43 generating oxazole peptidomimetics. The procedure is based on the instability under acidic conditions of the initially formed amino oxazole [226–227]. The modification includes a hydrolysis/activation/ cyclization sequence to provide 12–18-membered cyclic depsipeptides in good overall yields (upto 64%). As shown in Scheme 88, the process starts with a base catalyzed hydrolysis of the alkyl ester followed by protonation of the oxazole-scaffold to provide imminium cation **301**. Subsequent intramolecular cyclization induced by the carboxylate yielded the spiro-intermediate that via a nucleophilic attack of the tethered alcohol to the activated ester resulted in the macrocyclic depsipeptides **303**.

**Figure 4:**
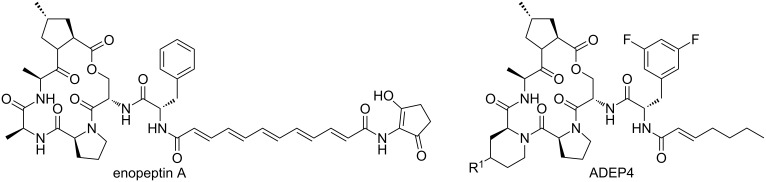
Enopeptin A with its more active derivative ADEP-4.

Another interesting procedure involves a MCR/macrolactonization strategy in order to obtain two structural derivatives of the in vivo active antibiotic acyldepsipeptide, ADEP-4 [228]. It is noteworthy that the pipecolic acid moiety in ADEP-4 enhances the in vitro and in vivo antibiotic activity as compared to its *N*-methylaniline precursor enopeptin A, thereby reflecting the importance of conformationally restricted elements (Figure 4).

**Scheme 89:**
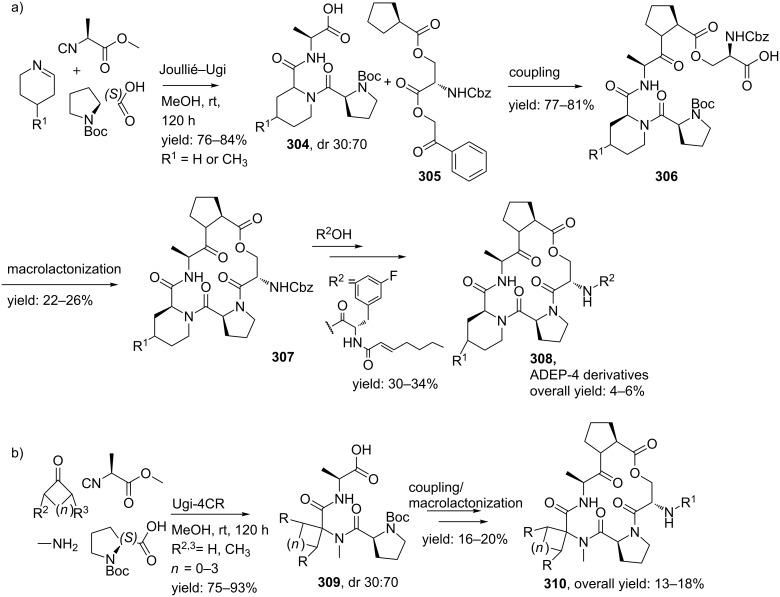
a) The Joullié–Ugi-approach towards ADEP-4 derivatives b) Ugi-approach for the α,α-dimethylated derivatives.

For these ADEP-4 derivatives, a Joullié–Ugi 3CR of cyclic imines, *N*-Boc-proline and isocyanopropanoate (derived from aniline methylester), followed by coupling with **305** and subsequent TFA-promoted saponification gave the macrocycle **307** (Scheme 89). Under these conditions the Ugi reaction proceeds in a diastereoselective fashion and for unclear reasons formation of the less active isomer (dr 30:70) was favoured. Chromatographic separation followed by an additional peptide coupling provided the final compounds **308** in 4–6% overall yield. In a variation an Ugi 4-CR was employed to construct α,α-dimethylated derivatives. Herein, the Ugi reaction of methylamine, *N*-Boc-proline, isocyanopropanoate and different ketones gave the desired tripeptides **309** in good to excellent yields (Scheme 89). The subsequent post-modification reactions provided macrocyclic analogues **310** in overall yields of 13–18%. To validate the antibacterial activity, the different scaffolds were screened against several drug-resistant bacterial strains, however, only the pipecolic derivatives showed antibacterial activity.

#### Macrocycles via MCR-Click-combinations

**Scheme 90:**
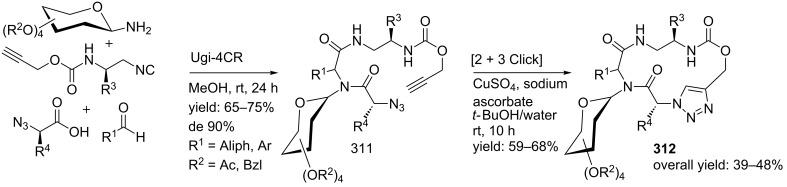
Ugi–Click-strategy for 15-membered macrocyclic glyco-peptidomimetics.

A third approach that can be utilized to construct macrocyclic peptidomimetics is a sequential Ugi–Click-combination. Via this approach the cyclic mimics are linked via a triazole-unit. Recently, Sureshbabu and co-workers used such a two-step sequence for a small library of 15-membered cyclic glyco-peptidomimetics [229]. In this approach, the Ugi reaction was performed with a variety of sugar-1-amines, aldehydes, azido acids and Poc-amino methyl isocyanide and afforded the linear Ugi-products as a mixture of two diastereomers in a ratio of 95:5. The Poc-group functions as protecting group in the Ugi reaction while being reactive in the subsequent Click reaction (Scheme 90). The Click reaction gave the macrocycles **312** in 39–48% overall yield. It is noteworthy that the cyclization was performed at mM concentrations to minimize dimerization processes.

**Scheme 91:**
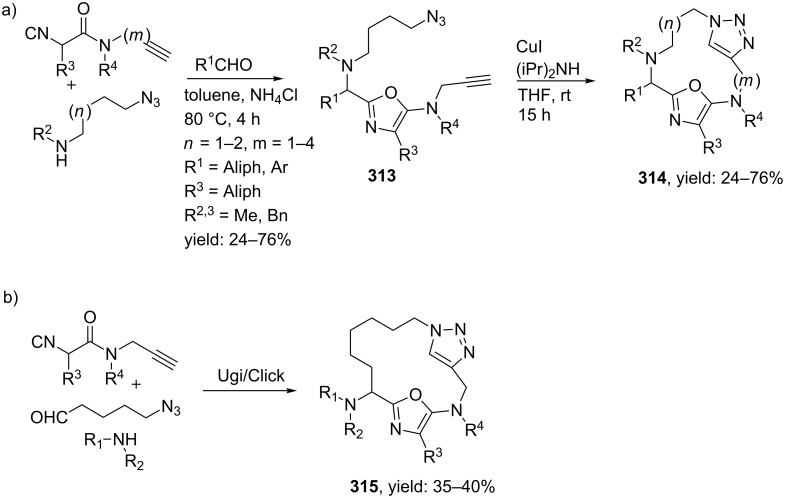
Ugi/Click combinations provided macrocycles containing both a triazole and an oxazole moiety.

In a variation to the aforementioned 5-amino-oxazole Ugi MCR/macrolactonization, the group of Zhu also synthesized macrocycles via a subsequent [3 + 2] cycloaddition reaction that contained both a triazole and an oxazole unit (Scheme 91) [230]. The Ugi reaction was performed in toluene and a variety of isocyanoacetamides and amines were employed as bifunctional inputs containing either a terminal alkyne or azide moiety. Herein, the isocyanoacetamides were derived from formylated amino acids via an azide-coupling and dehydration sequence. The subsequent cycloaddition was catalyzed by copper iodide, furnishing 14–16-membered macrocycles **314** in good yields (24–76%) that all contain a triazole (as amide biosteres) and an oxazole moiety (as dipeptide surrogate). In addition, it is worth noting that the reaction sequence could also be performed with tethered azide and alkyne moieties in the aldehyde and isocyanide inputs, thereby providing macrocycles with an *exo*-tertiary amine (**315**, 35–40%).

#### Macrocycles via MCR-S_n_Ar-procedures

**Scheme 92:**
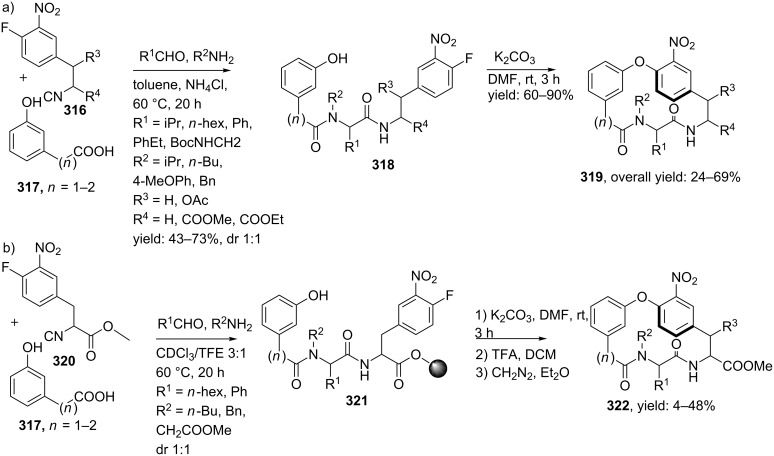
a) A solution-phase procedure towards macrocycles. b) Alternative solid-phase synthesis as was reported by Zhu et al*.* [238–239].

Macrocycles containing an aryl-ether bridge can be obtained via a combination of the Ugi reaction and a sequential aromatic substitution (S_n_Ar). In Nature, these particular macrocycles have been found and some of them possess interesting antibiotic [231–232], antitumor [233], or antifungal activities [234–235]. In addition, they are also found as potent ACE-inhibitors [236]. The most leading example is the antibiotic vancomycin, which found application as last remedy against multi-drug resistant microbial strains [237]. Zhu and co-workers designed both a solution and solid-phase synthesis of biaryl-ether-linked macrocycles (Scheme 92) [238–239]. In the solution-phase approach, they performed an Ugi reaction with a variety of aliphatic and aromatic amines and aldehydes, two acids and two isocyanides (synthetically derived) to arrive at the linear Ugi-products **318** in good yields (43–73%, dr 1:1). During the reaction no formation of Passerini-like or ammonia derived side products were observed. The solid-phase synthesis afforded the linear precursors by reacting resin-bound isocyanide **320** with a variety of different amines, aldehydes and acids in CDCl_3_/TFE [240]. The subsequent ring closure reaction was performed under basic conditions, providing the 16–17 membered macrocycles as two atropisomers for each diastereomer in moderate to high yields. Slightly better overall yields were observed for the solution-phase process (**319**, 24–69%), compared to the solid-phase route (**322**, 4–48%) [238].

**Scheme 93:**
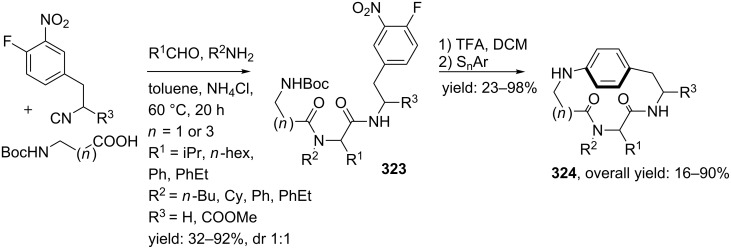
Ugi/cyclization towards cyclophane based macrocycles.

The same authors also published a similar solution-phase sequence towards cyclophane based-macrocycles having an aryl-*N*-alkyl bridge (Scheme 93) [241]. In this route the Ugi reaction was performed with aliphatic carboxylic acids, furnishing the dipeptides **323** in 32–92% yield. Subsequent TFA treatment followed by S_n_Ar-cyclization provided the 15-membered macrocycles **324** in 16–90% overall yield, again as two atropisomers.

#### Passerini-amine deprotection-acyl migration procedures

**Scheme 94:**
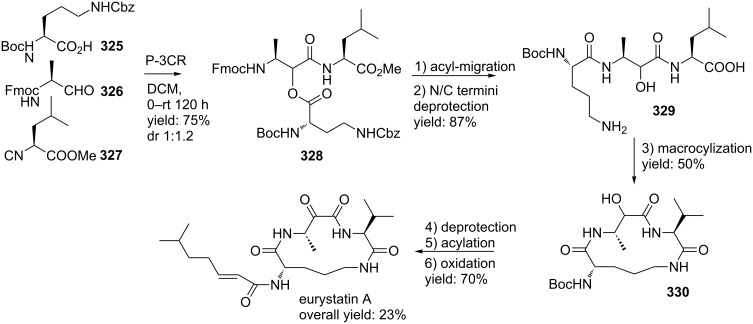
PADAM-strategy towards eurystatin A.

The Passerini-amine-deprotection-acyl migration (PADAM) strategy is a useful alternative to provide dipeptides that can be cyclized to the corresponding macrocycles via a subsequent peptide-coupling. The PADAM-strategy has been used for the total synthesis of eurystatin A and cyclotheanamide C, both are potent α-keto-amide-based protease inhibitors. Synthesis of eurystatin A was developed by Semple and co-workers and included *N*-protected amino acid **325,** enantiopure aldehyde **326** and leucine isonitrile **327** as MCR-substrates (Scheme 94) [242]. Subsequent *N*-Fmoc deprotection of the resulting linear adduct induced acyl-migration, in which deprotection and hydrolysis of the two N/C-termini provided the cyclization substrate **329**. A sequential *N*-Boc deprotection, acylation and oxidation provided the macrocyclic eurystatin A in 23% yield over 8 steps.

**Scheme 95:**
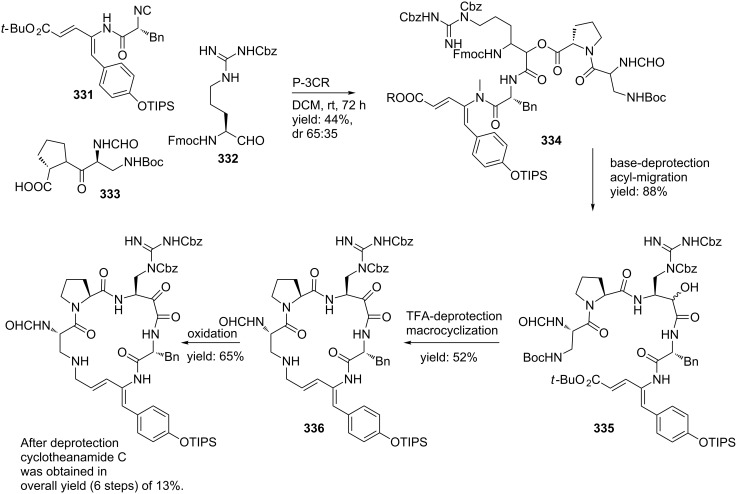
PADAM-approach for cyclotheanamide.

The PADAM-based synthesis of cyclotheanamide C was published by Aitken et al. (Scheme 95) [243]. In this report, the α-acyloxyamide precursor was obtained in 44% yield by reacting isocyanide **331** with a small excess of both Fmoc-amino aldehyde **332** and *N*-Boc dipeptide **333** (1.2 equiv). Again base promoted *N*-Fmoc deprotection induced acyl-migration, in which TFA treatment and subsequent peptide-coupling using TBTU/HOBt furnished macrocycle **336**. Final oxidation with DMP and base-catalyzed Cbz-removal gave the cyclotheanamide C-compound in 13% yield over 6 steps.

#### MCR-MCR cyclizations

**Scheme 96:**
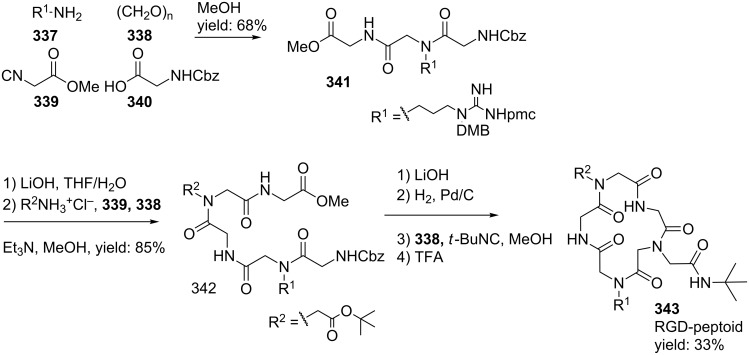
A triple MCR-approach affording RGD-pentapeptoids.

Even more challenging is the construction of macrocycles in which the cyclization step is also performed by a multicomponent reaction. Wessjohann and co-workers reported a combination of three sequential Ugi-MCRs towards RGD-pentapeptoids (Scheme 96) [244]. All three MCRs involved an Ugi-MCR, in which the first two provided linear products **341** and **342**, whereas the third MCR resulted in the ultimate macrocyclic peptoid structure. The final RGD-macrocycles **343** were obtained after TFA-deprotection in 33% overall yield. It was shown by the authors that a wide range of cyclic peptoids could be obtained by alternating the MCR-substrates. In particular, interchange of the amine component in the first two MCRs resulted in (retro)-peptoids consisting of a DGR-sequence.

A second powerful strategy to obtain macrocycles exclusively from combinations of multicomponent reactions is the MiB-approach (*m*ultiple multicomponent macrocyclizations *i*ncluding *b*ifunctional building blocks). Similar to the previous described MCR-MCR approach, macrocycles are in this approach obtained from two or more MCRs. However, in this particular process, the MCRs include two or more *unprotected* bifunctional groups such as diisonitriles, diamines or amino acids. The incorporation of these unprotected bifunctional substrates makes the construction of highly complex macrocycles even more straightforward and also allows scaffold diversification. In the literature, several Ugi or Passerini-based MiB-approaches have been reported and only two examples will be given in this review since they already have been extensively reviewed by the groups of Wessjohann and Rivera. For more details see also references [24,27–29,245–246].

**Scheme 97:**
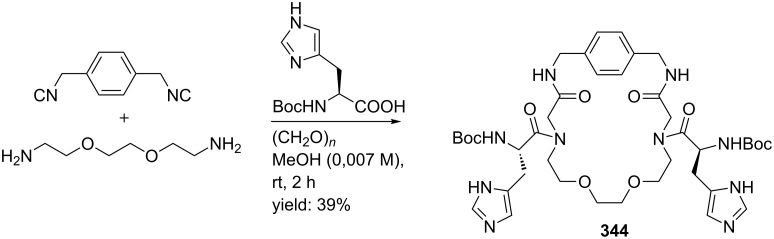
Ugi-MiBs-approach towards peptoid macrocycles.

An example of an Ugi-approach by Rivera and Wessjohann included symmetric diamines and diisonitriles in combination with formaldehyde and (protected) α-amino acids (Scheme 97). Via this procedure peptoid-based macrocycles **344** were obtained that contain biologically relevant side chains [245].

**Scheme 98:**
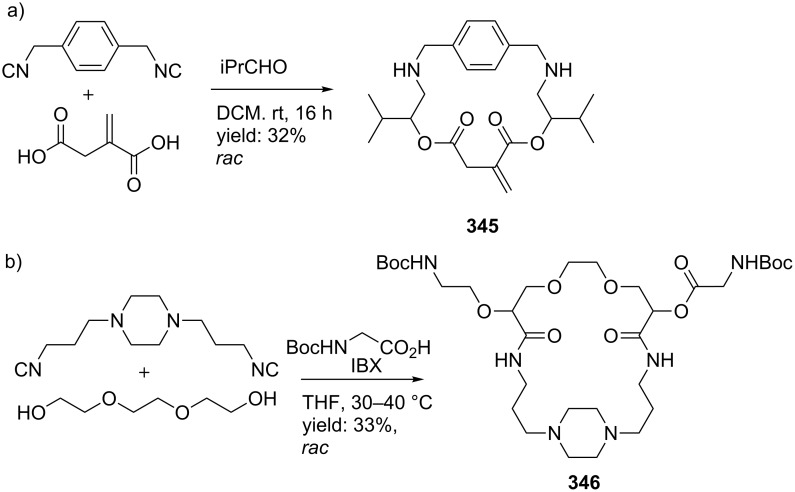
Passerini-based MiB approaches towards macrocycles **345** and **346**.

The same group also reported a Passerini-based MiB-approach (Scheme 98) [247]. The multicomponent reactions were either performed with diacid/diisonitrile combinations or with diisonitrile/dialdehyde bifunctional groups, providing the macrocycles **345** and **346** in 32% and 33% yield, respectively. It was shown that the latter combination requires in situ-generation of the dialdehydes from dialcohols via an oxidative Passerini reaction. One reason for this in situ generation was the acid-instability of aldehydes [248].

**Scheme 99:**
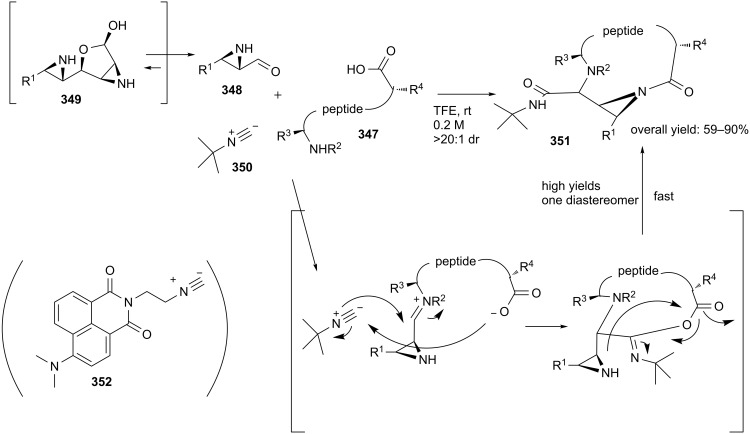
Macrocyclic peptide formation by the use of amphoteric aziridine-based aldehydes.

Finally, Yudin et al. [219,249] developed interesting and very effective strategies to construct macrocyclic peptidomimetics through an MCR-induced cyclization. Their approach includes macrocyclization of peptides of type **347** using so-called amphoteric aziridine-based aldehydes **348** (used as the corresponding dimer **349**) in combination with isocyanides **350** (Scheme 99). As became clear from discussions in this review, the use of the Ugi reaction in a “traditional” sense to induce macrocyclization of peptidic α-amino acids (as bifunctional inputs), aldehydes and isocyanides usually produces mixtures of diastereoisomers of macrocyclic peptides and requires high dilution conditions to minimize overall dominant formation of cyclo di- or oligomerization products. The amphoteric aldehydes **348** in the Yudin macrocyclization strategy can be applied under conventional reactant conencentrations and overcomes both the stereoselectivity and oligomerization issues producing in good yields and excellent diastereoselectivities macrocyclic peptides **351** avoiding epimerization. In follow-up work a number of interesting applications were discussed [250] including the use of solvatochromic isocyanides **352** to access cell permeable macrocyclic peptide vectors [251] and RGD fluorescent probes [252]. In addition, the macrocyclization tool proved very useful in the exocyclic control over turn induction in macrocyclic petides [253].

## Conclusion

Isocyanide-based multicomponent reactions in combination with subsequent cyclization reactions have become valuable tools in the design and synthesis of cyclic constrained peptidomimetics. These IMCRs give rapid access to these complex target molecules from relatively simple starting materials addressing both structural diversity and complexity. Furthermore, after the initial MCRs several post-cyclization condensations can be utilized, since a variety of unreactive or monoprotected functionalities are tolerated in the initial MCRs. This ultimately leads to cyclic constrained peptidomimetics in only a few steps as compared to often much longer more traditional sequential procedures.

We discussed many examples of four to seven membered (bi)cyclic dipeptide isosteres such as lactams, triazoles, oxazoles and thiazoles that can be easily incorporated via these IMCR/cyclization protocols, in which it was even possible to provide triazole based peptidomimetics. Incorporating dipeptide mimics into peptides introduces conformational order, improves stability against enzymatic degradation and allows the peptidic structures to adopt secondary structures such as turns and α-helices. A nice example of this was the bicyclic diketopiperazines as they perfectly force the peptide-like structure into a type I β-turn.

In addition, the IMCR/cyclization strategies have also shown to be highly suitable for the synthesis of macrocyclic peptidomimetics. The interest for macrocycles is based on two important properties. These macrocyclic peptoids combine conformational order with flexibility and they are stable against terminal group degradation by proteases. As discussed here, the IMCR-based synthesis of such macrocycles usually requires a subsequent head-to-tail cyclization such as ring-closing-metatheses, cycloadditions, lactonizations, peptide-couplings or nucleophilic substitutions. Even more interestingly is the synthesis of cyclic peptidomimetics via multiple MCRs as was described for macrocyclic RGD-peptoids. These multiple MCR-approaches have the advantages to also introduce structural diversity and complexity at the cyclization step.

However, still a main flaw of MCR-based strategies for the synthesis of constrained peptidomimetics is the often poor stereocontrol in these reactions. This is crucial since optically pure peptide-like products are essential for proper study of peptide-peptide interactions, therefore, for the future of this chemistry in this field the design of (dia)stereoselective multicomponent reactions is highly desirable. Several research groups have indeed realized this challenge and are developing such asymmetric multicomponent reactions. Perhaps a prominent example is the bio-catalytic synthesis of optically active pyrrolidines that can be used in a MCR-based synthesis of telaprevir. Further developments may rely on the use of these asymmetric approaches and will make the multicomponent reaction an even more useful tool for the design of novel conformationally constrained peptidomimetics.
